# Strength enhancement and slip behaviour of high-entropy carbide grains during micro-compression

**DOI:** 10.1038/s41598-019-46614-w

**Published:** 2019-07-15

**Authors:** Tamás Csanádi, Elinor Castle, Michael J. Reece, Ján Dusza

**Affiliations:** 1 Institute of Materials Research, Slovak Academy of Sciences, Watsonova 47, 04353 Košice, Slovak Republic; 2 School of Engineering and Material Science, Queen Mary University of London, Mile End Road, London, E1 4NS UK

**Keywords:** Characterization and analytical techniques, Ceramics

## Abstract

Bulk polycrystalline high-entropy carbides are a newly developed group of materials that increase the limited compositional space of ultra-high temperature ceramics, which can withstand extreme environments exceeding 2000 °C in oxidizing atmospheres. Since the deformability of grains plays an important role in macromechanical performance, in this work we studied the strength and slip behaviour of grains of a spark-plasma sintered (Hf-Ta-Zr-Nb)C high-entropy carbide in a specific orientation during micropillar compression. For comparison, identical measurements were carried out on the monocarbides HfC and TaC. It was revealed that (Hf-Ta-Zr-Nb)C had a significantly enhanced yield and failure strength compared to the corresponding base monocarbides, while maintaining a similar ductility to the least brittle monocarbide (TaC) during the operation of ${\boldsymbol{\{}}{\bf{110}}{\boldsymbol{\}}}{\boldsymbol{\langle }}{\bf{1}}\bar{{\bf{1}}}{\bf{0}}{\boldsymbol{\rangle }}$ slip systems. Additionally, it was concluded that the crystal orientation and stress conditions determine the operation of slip systems in mono- and high-entropy carbides at room temperature.

## Introduction

The development of materials for engineering applications exceeding 2000 °C in oxidizing atmospheres, such as hypersonic vehicles and spacecraft, is a great challenge for material scientists. To date, ultra-high temperature ceramics (UHTCs) are the only, and limited, group of materials that can withstand such extreme environments. They are based on the refractory borides, carbides and nitrides of the group of IV and V transition metals and are typically defined as having melting temperatures higher than 3000 °C^1^, with HfC exhibiting the highest melting point of all materials (4232 ± 84 K)^2^ known to man. Due to their strong bonds, UHTCs and their composites possess an unusual set of properties, including high hardness (>20 GPa) and strength (>500 MPa) with excellent oxidation resistance and good resistance to thermal shock even at temperatures exceeding 2000 °C^3–5^. For ultra-high temperature applications, such as rocket propulsions, hypersonic and re-usable atmospheric re-entry vehicles, UHTCs are the only suitable materials that can withstand or protect components exposed to extreme environments. As these developing technologies become more advanced and more demanding, UHTCs are coming under increasing pressure to perform under even more extreme operating conditions. A greater selection of UHTCs that exhibit a much broader range and combination of physical, chemical and mechanical properties are therefore required to meet these demands. A promising way to overcome this problem is the exploration of a new class of materials, motivated by the discovery of high-entropy alloys^6,7^ and pioneering work on entropy-stabilized oxides^8^. These so-called, bulk ‘high-entropy UHTCs’, which are composed of four or five or more different transition metal elements of equimolar proportions and boron or carbon atoms, form hexagonal or cubic solid solution structures, respectively. In these ceramics, the arrangement of atoms at metallic sites may be completely random, similar to high-entropy metal alloys. It was shown that single phase crystalline materials can be formed because of their enhanced molar configurational entropy (${\rm{\Delta }}{S}_{mix}=RlnN$, where *N* is the number of equimolar components and *R* is the gas constant) which stabilizes the solid solutions^9^. Recently, these bulk high-entropy UHTCs have been successfully synthesized both from metal diborides^10^ by Gild el al. and from metal carbides^11^ by the present authors. Primary property assessments of these novel materials suggests that high-entropy ultra-high temperature ceramics have the potential to broaden the compositional space beyond the current limited set of UHTCs, and that their hardness could go beyond those of the base borides^10^ and carbides^11–13^.

Regarding high-entropy carbides, understanding the factors that influence their mechanical properties, including a comparison with the base transition metal monocarbides (HfC, TaC, ZrC, NbC, VC and TiC), is essential to their further development. From the viewpoint of macro-scale applications, their low ductility is a key-property that might be a drawback in such high hardness materials. Therefore effort should be devoted to understanding the deformation behaviour of high-entropy carbides, including the identification of the slip systems operating within grains at the micro-scale; which is rather limited in the base monocarbides, and can only be tested practically using indentation techniques. It was revealed that in the group of IV and V transition metal carbides (TMCs), which exhibit a cubic rock salt crystal structure (space group Fm-3m, No. 225), slip is preferred on $\{110\}\langle 1\bar{1}0\rangle$ type slip systems at low temperature (e.g. 77 K for TaC) due to strong metal-carbon (covalent-ionic) bonds. With increasing temperature the bonds tend to be less directional forming more metallic like character and slip becomes more similar to fcc metals, namely $\{111\}\langle 1\bar{1}0\rangle$ type, through a brittle to ductile transition where both slip system families are active. At room temperature, group IV TMCs (TiC, ZrC, HfC) were reported to slip on $\{110\}\langle 1\bar{1}0\rangle$ and be more brittle than group V monocarbides (VC, NbC, TaC) which slip on $\{111\}\langle 1\bar{1}0\rangle$^14–17^. Recently, first-principles density functional theory (DFT) calculations revealed that the operation of the $\{111\}\langle 1\bar{1}0\rangle$ slip systems in group V monocarbides is due to the possibility of the formation of intrinsic stacking faults by dissociation into partial dislocations with Burgers vector $\frac{a}{6}\langle 112\rangle$, which requires only slightly higher stresses than the activation of $\{110\}\langle 1\bar{1}0\rangle$ type slip^17,18^. To enhance ductility, experimental data shows that the transition from $\{110\}\langle 1\bar{1}0\rangle$ to $\{111\}\langle 1\bar{1}0\rangle$ slip systems can be promoted by increasing carbon content (loss of carbon vacancies) in substoichiometric compositions of group V monocarbides^15,16^ and by the presence of other solute metallic atoms (substitutionals) in the group IV TMC lattice^19^. Contrary to substoichiometric compositions of group IV monocarbides, which show a marked decrease in hardness, in the (Hf-Ta)C binary carbides the operation of $\{111\}\langle 1\bar{1}0\rangle$ slip systems is accompanied by a hardness enhancement. In addition to the chemical composition, the orientation of the grains and the testing method also have a significant influence on slip operation as shown for TaC and ZrC during micropillar compression^14,15,17,20^.

As the demand increases for UHTCs with improved macromechanical properties, the development of a new range of high performance high-entropy carbides should be possible if a better understanding of their deformation behaviour at the micro-scale can be obtained. The yield and failure strength of the grains and operation of slip systems in them will play a key-role in their ductility at the macro-scale. Here, we show how a newly synthesized (Hf-Ta-Zr-Nb)C high-entropy carbide has a significant enhancement of yield and failure strength in micropillar compression tests compared to the corresponding base monocarbides; and how the crystal orientation and stress conditions determine the operation of slip systems at room temperature.

## Results

### Bulk (Hf-Ta-Zr-Nb)C high entropy carbide and micropillars

**Figure 1:**
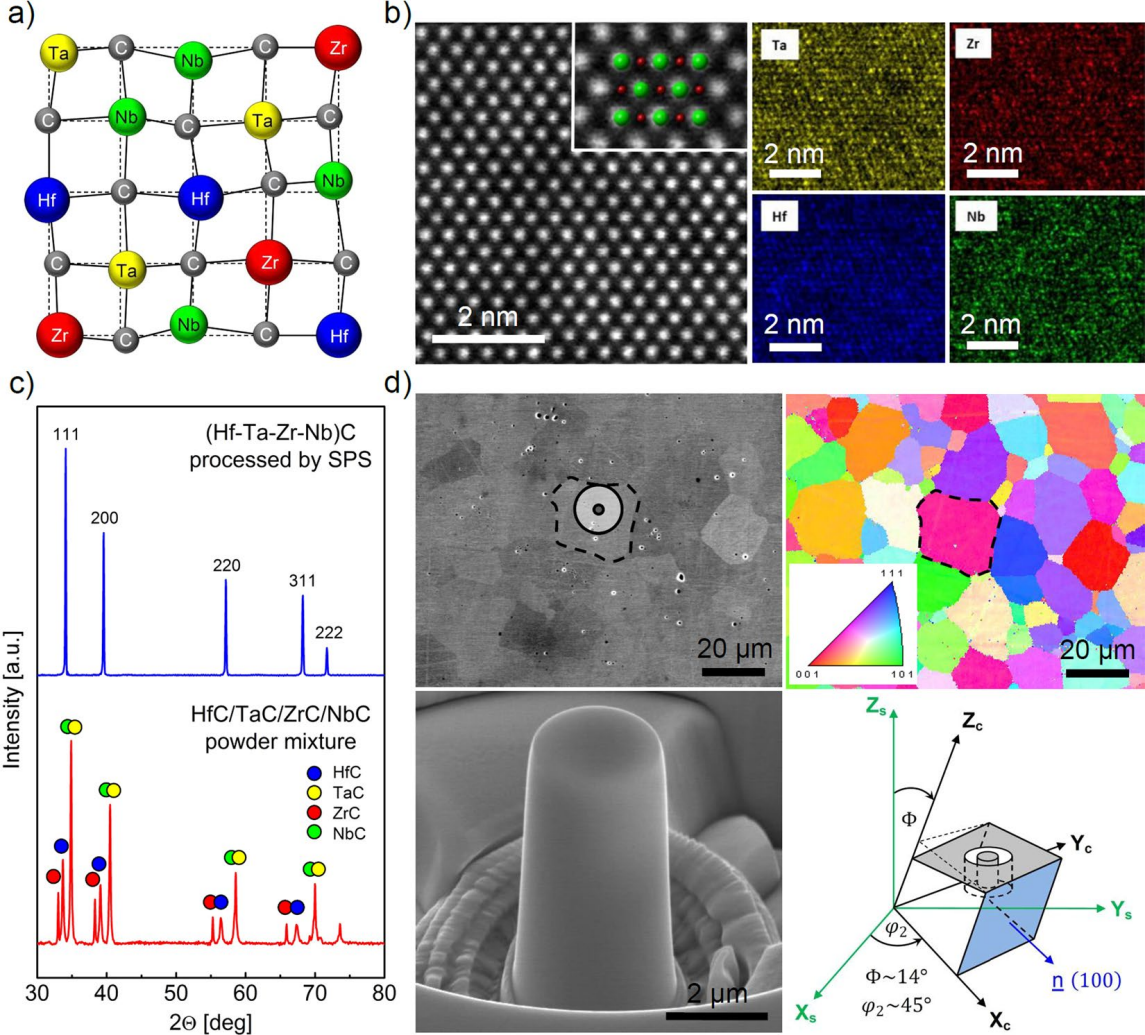
Structure of the (Hf-Ta-Zr-Nb)C high-entropy carbide at atomic, micro- and macroscale. (**a**) Schematic of an ideal lattice structure of the high-entropy carbide, showing lattice distortions due to different valence electron concentration and size of transition metal atoms. (**b**) HAADF STEM micrograph of (Hf-Ta-Zr-Nb)C along the [011] zone axis (Fm-3m) and atomically resolved EDS maps, showing random distribution of constituent elements. The magnified inset indicates the atomic positions of Hf, Ta, Zr or Nb (green) and C (red). (**c**) XRD data for the ball milled HfC/TaC/ZrC/NbC powder mixture and the (Hf-Ta-Zr-Nb)C sample after sintering by SPS. (**d**) SEM of microstructure of (Hf-Ta-Zr-Nb)C, corresponding EBSD map and a FIB-milled micropillar located in a selected grain marked on both the SEM and EBSD images. Micropillars were fabricated in grains of a specific orientation (Φ ~ 14° and φ_2_ ~ 45°) as shown in the schematic.

The investigated UHTC materials were bulk single phase polycrystalline HfC, TaC monocarbides and a (Hf-Ta-Zr-Nb)C high-entropy carbide. We processed the compositions by ball-milling and spark-plasma sintering (SPS), applying a two-step heating profile with a 10 min dwell at 1800 °C and 7 min dwell at 2300 °C, as reported in our recent paper^11^. In this section, we look at the results obtained on the structure of (Hf-Ta-Zr-Nb)C at the macro, micro and atomic levels, which is necessary to understand the micropillar compression results described later; information on the structures of HfC and TaC can be found in the Supplementary data (Supplementary Fig. 1). A schematic of an ideal lattice structure for (Hf-Ta-Zr-Nb)C is show in Fig. 1a. The lattice distortion is exaggerated and its degree might be different for the different possible metal-metal and metal-carbon pairs. Although we characterised (Hf-Ta-Zr-Nb)C using various techniques^11,21^, the lattice structure has not yet been completely characterised. The difference in atomic size^11^ and valence electron concentrations (VEC) of the transition metals^22^ in the HEC are thought to have a simultaneous lattice distorting effect, based on the works done on high-entropy metallic alloys^23^ and high-entropy oxides^24^. Considering that the covalent-ionic metal-carbon bonds are the strongest ones in (Hf-Ta-Zr-Nb)C and the smallest atomic radius belongs to carbon in the system, it might be that the position of the metal sites are slightly perturbated and the carbon atoms are arranged in a distorted sub-lattice that accommodates the different atomic sizes and VECs of the metals, similar to what has been reported for entropy-stabilized oxides^24^. This lattice distortion is assumed to hinder dislocation motion in a given slip system compared to the corresponding monocarbide lattice. In addition to lattice distortion, VEC seems to be the principal indicator that controls the metallic character of high-entropy carbides, resulting in higher ductility for (Hf-Ta-Zr-Nb)C (VEC = 8.5) compared to HfC (VEC = 8) since a larger is VEC is associated with higher ductility for rocksalt structured carbides^22^. The effective VEC of 8.5 is expected for (Hf-Ta-Zr-Nb)C according to its equiatomic composition, similar to the binary (Hf-Ta)C^19^. Therefore, the VEC of (Hf-Ta-Zr-Nb)C is assumed to have a dual role: 1) increasing effective VEC improves the metallic character of high-entropy carbides, at least compared to HfC, improving their ductility, 2) the difference in VEC of metals and associated differences in lattice distortion, producing strengthening and higher work hardening rates in high entropy carbides compared to the monocarbides.

At the atomic level, the random arrangement of TMCs in the (Hf-Ta-Zr-Nb)C has been verified by energy dispersive X-ray spectroscopy (EDS) maps of a high angle annular dark field scanning transmission electron microscopy (HAADF STEM) image taken along the [011] zone axis as shown in Fig. 1b. The magnified inset in the HAADF STEM micrograph indicates the atomic positions of Hf, Ta, Zr or Nb elements in green and carbon in red. At the macro level, the crystalline and single phase structure of (Hf-Ta-Zr-Nb)C has been confirmed by X-ray diffraction as shown in Fig. 1c. The comparison of XRD data collected from both the ball milled HfC/TaC/ZrC/NbC mixture and the high-entropy carbide sample after sintering by SPS shows the individual peaks for the powder mixture and only one set of peaks for (Hf-Ta-Zr-Nb)C, suggesting complete inter-diffusion between the different TMC elements to produce a single, multi-metal fcc carbide phase. Figure 1d shows the micro-scale structure of the high-entropy carbide using scanning electron microscopy (SEM) and the corresponding electron backscatter diffraction (EBSD) images, together with a focused ion beam (FIB) milled micropillar, which is located in a selected grain marked on the SEM and EBSD images. The grain size varies approximately in the range of 5–30 μm with an average grain size of 12 μm, and there are only small pores present in the microstructure with diameter up to approximately 2 μm. The crystallographic orientation of the individual grains is distributed randomly. For further details the reader is referred to the authors’ recent works^11,21^.

Four micropillars were fabricated in visibly pore free regions of large (Hf-Ta-Zr-Nb)C grains of a specific orientation (Φ ~ 14° and φ_2_ ~ 45°) according to the schematic in Fig. 1d. The reason for this grain selection of the above defined orientations is that the Schmid factor is equal for both the $\{110\}\langle 1\bar{1}0\rangle$ and $\{111\}\langle 1\bar{1}0\rangle$ slip systems, as explained in detail in the Discussion section. Thus, it is easy to determine from one set of measurements which slip system family has a lower critical resolved shear stress under the uniaxial stress conditions applied to the high-entropy carbide grains/single crystals. Similar to the high-entropy carbide, identical microstructure analyses and micropillar fabrication process were applied to the HfC and TaC samples. Corresponding SEM, EBSD maps and SEM of micropillars can be found in the Supplementary data.

### Micropillar compression of mono- and high-entropy carbides

**Figure 2:**
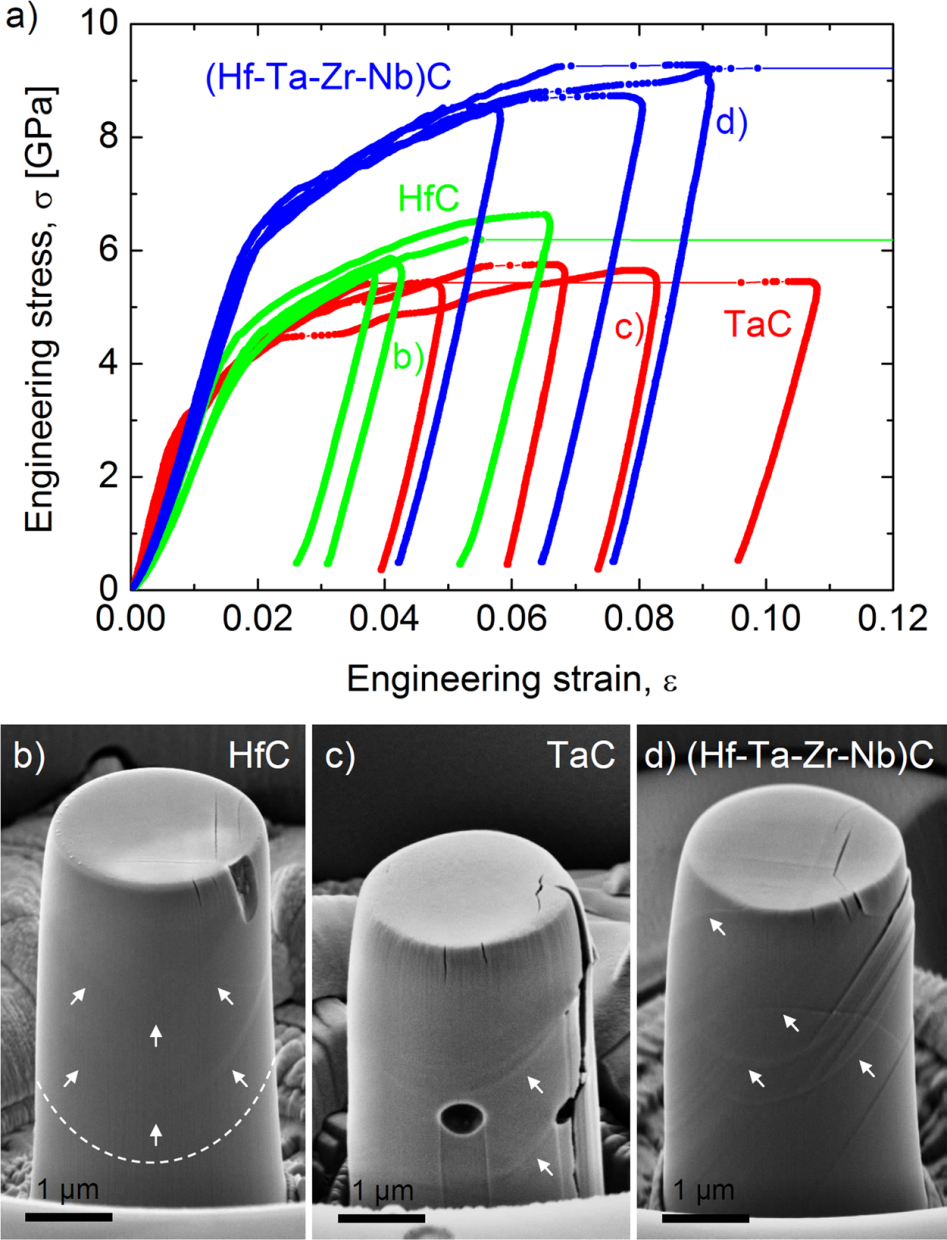
Micropillar compression of mono- and high-entropy ultra-high temperature carbides. (**a**) Characteristic stress-strain curves obtained during micropillar compression of individual grains of HfC, TaC and (Hf-Ta-Zr-Nb)C, showing an enhanced yield and failure strength for the high-entropy ultra-high temperature carbide. Micropillars of similar orientations (Φ ~ 14°) exhibit limited plasticity for grains of (**b**) HfC but more ductile behaviour for (**c**) TaC and (**d**) (Hf-Ta-Zr-Nb)C high-entropy carbide. Slip traces on micropillar surfaces are marked by arrows.

Engineering stress-strain curves obtained during micropillar compression of HfC, TaC and (Hf-Ta-Zr-Nb)C grains of orientation Φ ~ 14° and φ_2_ ~ 45° are shown in Fig. 2a. Despite the macroscopically brittle nature of the ultra-high temperature monocarbides, their micro-scale behaviour shows considerable plasticity before their final failure. The yield points, including strains and stresses, were characteristic for each material tested due to their low scatter. Evidence of the plastic behaviour in the micropillars in the form of slip traces was found for all of the materials as shown in Fig. 2b–d, similar to that reported for micro-compression of oriented single crystals of TaC by Kiani *et al*.^20^. In our work, the micropillars were compressed until a rapid increment in displacement occurred at a given load or the depth was in the region where some of them have collapsed, achieving a total deformation of ε_HfC_ ~ 0.06, ε_TaC_ ~ 0.1 and ε_HEC_ ~ 0.08 for HfC, TaC and (Hf-Ta-Zr-Nb)C, respectively. Most of the micropillars were unloaded before their final fracture, making it possible to perform further SEM investigations into their deformation behaviour. Some of the pillars collapsed during compression, as shown by horizontal lines in Fig. 2a. In order to compare the ductility of HfC, TaC and (Hf-Ta-Zr-Nb)C, the occurrence of rapid increments in displacement were also considered despite the limited number of micropillars that were tested up to their collapse. This suggests wider plastic deformability of (Hf-Ta-Zr-Nb)C compared HfC and similar ductility to the least brittle monocarbide (TaC). Micro-compression curves show an initial linear elastic region, followed by plastic flow at different strain and stress levels characteristic to each material; as listed in Table 1. The lowest yield stress corresponds to TaC at 3.03 ± 0.11 GPa and is slightly higher for HfC with a value of 3.93 ± 0.28 GPa as shown in Fig. 2a. In the case of the high-entropy carbide, a considerable yield strength enhancement was found with a value of 6.20 ± 0.12 GPa. This is about 60% higher than that of HfC, which is the hardest base monocarbide material^11^. Interestingly, the yield strength enhancement is much larger than that which was measured from their nanohardness in our recent work, as listed in Table 1. This is assumed to be related to the different stress fields in micropillar compression and indentation, which is analysed further in the Discussion section.

Regarding the plasticity of the tested materials, HfC is the most brittle, showing hardly any discernible slip patterns on the surface of the micropillars before their collapse; which are marked in Fig. 2b. In the case of TaC, the slip lines on the micropillars are more visible and plastic deformation could be maintained without final failure, despite the presence of pores, which act as stress raisers (Fig. 2c**)**. The more ductile behaviour of the TaC against HfC is in agreement with earlier hardness measurements and is thought to be related to differences in slip activation^14–17^. Regarding (Hf-Ta-Zr-Nb)C, in addition to its considerably enhanced yield stress, it exhibits larger work hardening than that of HfC and shows similar ductility to TaC with more pronounced slip patterns observed on the micropillars (Fig. 2d**)**. This suggests that the nature of the bonding in the high-entropy carbide is similar to TaC, showing a more metallic character than that of HfC. Following the strain hardening of the micropillars, caused by the interaction of dislocations, cracking occurred mostly on their top surface and propagated down the pillar axis leading to their final collapse. The enhanced strength and ductility of the (Hf-Ta-Zr-Nb)C confirms unambiguously that high-entropy carbides open up a new compositional space for UHTCs with mechanical properties that potentially can exceed those of the base carbides. A complete understanding of their deformation behaviour is essential to their further development. Thus, the operation of the slip systems and cracking is analysed in the following section.

### Slip systems and cracking during micro-compression

**Figure 3:**
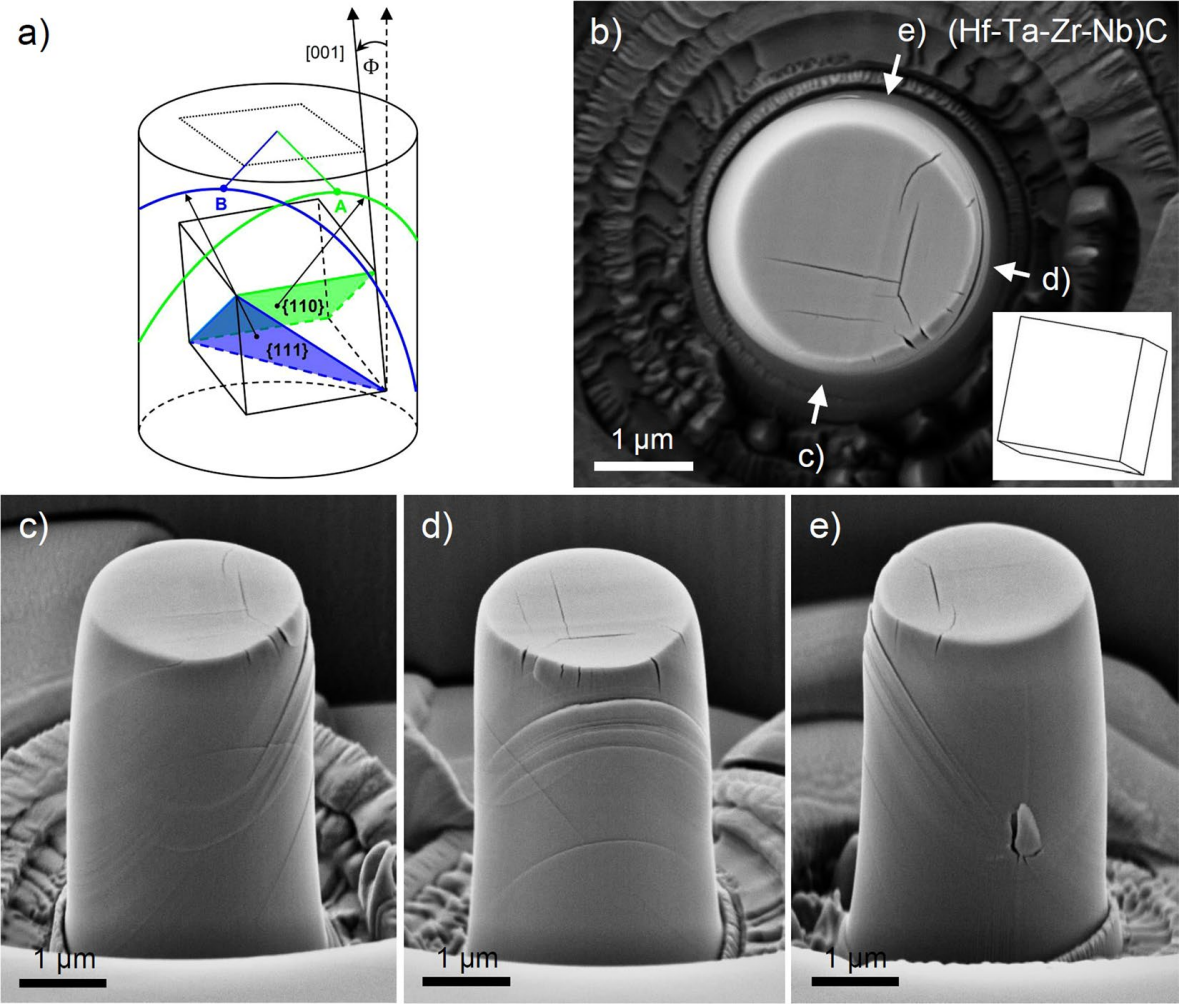
Determination of activated slip systems in high-entropy ultra-high temperature carbide. (**a**) Slip patterns expected to be formed on pillar surface during the operation of {110} and {111} type slip planes. (**b**) Top view of a compressed (Hf-Ta-Zr-Nb)C micropillar with the inset of the corresponding crystal orientation determined by EBSD. (**c**–**e**) Side view images of the compressed micropillar rotated by 90° relative to each other as shown in Fig. 3b).

Analysis of the active slip systems is described for the example of (Hf-Ta-Zr-Nb)C due to its more pronounced slip patterns compared to TaC and HfC. The slip analysis of the HfC and TaC micropillars are shown in the Supplementary Figs 2 and 3. It important to note that all of the three compressed high-entropy micropillars, which remained in one piece, exhibited similar slip patterns to that which was selected as an example. To easily distinguish between the $\{110\}\langle 1\bar{1}0\rangle$ and $\{111\}\langle 1\bar{1}0\rangle$ slip systems, slip patterns of the corresponding slip planes were analyzed on the surface of micropillars as shown in Fig. 3a. Based on this scheme, there is a difference in the orientation of the slip traces, which have an inclined ellipsoidal shape, relative to the crystal position during the operation of {110} and {111} type planes. In the case of the activation of the {110} planes, represented in green, the projection of the {100} planes on top of the micropillar is visible from the viewpoint of the maximum (or minimum) point (denoted by A) of a selected slip line. This is approximately the <100> direction of the crystal lattice since it has only a small tilt of Φ ~ 14°. In the case of the operation of {111} planes, indicated in blue, the maximum of the slip patterns (denoted by B) is located approximately along the viewpoint of the <110> direction of the lattice.

Regarding the top view SEM image of (Hf-Ta-Zr-Nb)C, cracks were visible approximately parallel with the $\langle 001\rangle$ traces of the crystal lattice, measured by EBSD, as shown in Fig. 3b. Thus, the side view SEM images were taken along approximately the <100> directions indicated in Fig. 3b. In all of the side view SEM images (see Fig. 3c,d), it is clearly visible that the maximum of the slip lines (point A) are located approximately along the <100> directions, confirming unambiguously the operation of {110} slip planes. Further analysis of the side view SEM images shows that several different {110} type planes were activated and there was one dominant set which caused a visible side shift along the direction from the viewpoint of d) in Fig. 3b. This suggests that the slip direction has a component that is approximately parallel with the [100] or [010] directions. Taking into account that micro-compression requires also a component of slip direction parallel with vertical (approximately [001]) direction, the only possible low index slip direction that satisfies these conditions is the <110> type, which is reported commonly for transition metal carbides^14–17^. The above described slip analysis led to the same results for TaC and HfC (Suppl. Figs 2 and 3). Thus, the operating slip systems in mono- and high entropy carbide grains of orientation of Φ ~ 14° and φ_2_ ~ 45° is the $\{110\}\langle 1\bar{1}0\rangle$ under uniaxial stress conditions. This result is unexpected since (Hf-Ta-Zr-Nb)C and TaC exhibited more ductile behaviour than HfC; and TaC is commonly reported to slip on the $\{111\}\langle 1\bar{1}0\rangle$ slip systems during indentation.

**Figure 4:**
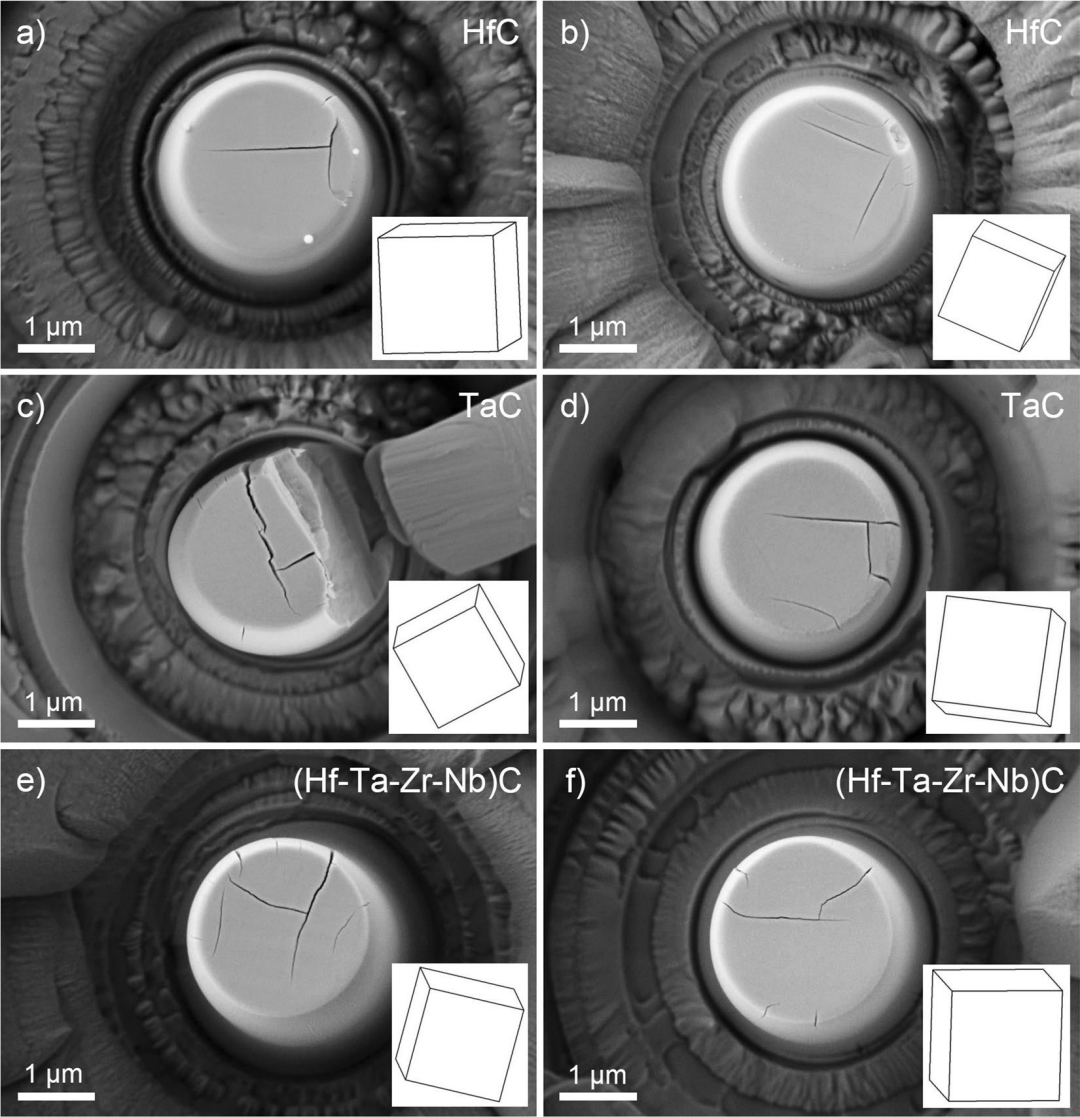
Cracking in mono- and high-entropy ultra-high temperature carbides. Crack paths are parallel with {100} type crystal facets of insets, determined by EBSD, on top surface of compressed (**a**), (**b**) HfC, (**c**), (**d**) TaC and (**e**), (**f**) (Hf-Ta-Zr-Nb)C micropillars.

**Figure 5:**
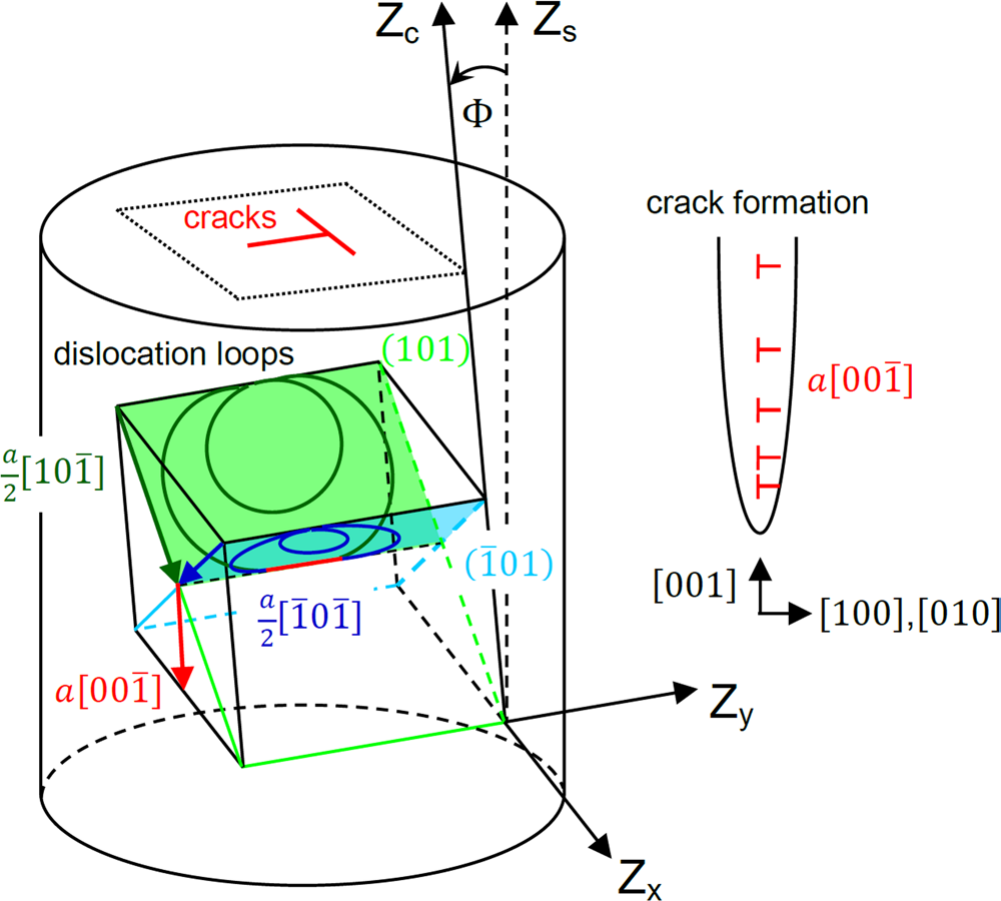
Schematic of cracking in mono- and high-entropy ultra-high temperature carbides. Cracks paths on the centre of top surface of the micropillars are assumed to cause by the interaction of dislocation loops (denoted by red) on opposite {110} type planes via the formation of $a[00\bar{1}]$ dislocations that pile up along (100) and (010) planes.

In addition to the slip operation, cracking also occurred on the top surface of the compressed micropillars, as shown for HfC, TaC and (Hf-Ta-Zr-Nb)C in Fig. 4. In all of the cases, the majority of the cracks are located on the top of the micropillars, which suggests that the cracks are initiated where the stress is highest due to the lowest pillar diameter. Some of the cracks started presumably on pre-existing defects on the top surfaces (e.g. pores, unintentional cuts by FIB, etc.). The misalignment between the tip and the micropillar, which is unavoidable in *ex-situ* testing, could initiate also cracks on one side of the micropillars’ edge that start to propagate inward to the pillar as shown in Fig. 4d–f. The above mentioned effects on cracking were not analyzed in detail because they are less significant than the crack paths that are visibly parallel with the {001} facets on the top of the micropillars (see cracks along $\langle 001\rangle$ traces). The influence of pores and FIB machining on cracking is considered to be negligible compared to the inherently brittle nature of transition metal carbides. This is based on our earlier works on ZrB_2_ grains, which did not show a high degree of cracking along their prismatic direction during micro-compression^25,26^, although the conditions of preparation and testing were identical to the present work. Cracks parallel with the {001} facets are located close to the centre of the micropillars and their formation is assumed to be due to the interaction of dislocations on different slip planes; similar to the work reported by Rowcliffe and Hollox on the indentation of TaC^27^. In our work, dislocations begin to slip on the $\{110\}\langle 1\bar{1}0\rangle$ systems at the top of the micropillars where stress is the highest due to their tapering. Considering two intersecting slip bands on the (101) and $(\bar{1}01)$ planes, the propagation of dislocation loops, with corresponding Burgers vectors of $\frac{a}{2}[10\bar{1}]$ and $\frac{a}{2}[\bar{1}0\bar{1}]$ along these planes, leads to their interaction according to the below equation as shown in Fig. 5.

1 $$\frac{a}{2}[10\bar{1}]+\frac{a}{2}[\bar{1}0\bar{1}]\to a[00\bar{1}]$$

Taking into account that the energy of dislocations is proportional to the square of their lengths, the formation of $a[00\bar{1}]$ dislocations is energetically favourable. However, the $a[00\bar{1}]$ dislocations are immobile since they do not lie in the (101) and $(\bar{1}01)$ planes. This results in their pile up along (100) plane, and due to similar reasons along the (010) plane, leading to cracking, with crack paths approximately parallel with these planes on the top surface of the micropillars (see Fig. 5) in agreement with experiments in Figs 3b and 4. These cracks can propagate downward along the axis of the micropillars during compression, leading to their fracture as indicated for TaC in Fig. 4c.

The formation of crack paths along the {100} planes is in agreement with DFT simulations of cleavage energies and strengths, which predict that the lowest value is for the {100} planes in stoichiometric cubic carbides^28^. Preference of fracture along {100} planes among the low index planes was explained by the lowest number of metal-metal and metal-carbon bonds that need to be broken^28^. This work revealed that carbon content has a significant influence on the cleavage planes, resulting in the lowest energy for {111} planes in substoichiometric carbides when carbon atoms are depleted from these planes.

## Discussion

To analyse the elastic behaviour of the micropillars, the measured Young’s modulus values of oriented micropillars were compared with theoretical predictions for HfC and TaC based on the corresponding DFT simulated elastic constants by Smith *et al*.^19^. It resulted in a monotonically decreasing Young’s modulus rotating the loading directions from [001] to $[11\bar{1}]$, ranging from about 500–420 GPa and 600–440 GPa for HfC and TaC, respectively. From the $[11\bar{1}]$ orientation to the [110] orientation a slight increase was found for HfC and TaC with values in the range of 420–440 GPa and 440–470 GPa, respectively. The calculated Young’s modulus (*E*_calc_) of the micropillars with orientation of Φ ~ 14° and φ_2_ ~ 45° is within 6% of the experimental results $(\frac{{\rm{\Delta }}E}{E}=\frac{{E}_{calc}-E}{{E}_{calc}})$ as indicated in Table 1, confirming the accuracy of the measurements and the single crystalline structure of the micropillars. The comparison of the Young’s modulus values with the indentation modulus reported in our recent work^11^ are not discussed as those are not directly comparable due to the elastic anisotropy mentioned above. The Young’s modulus characterizes the elastic behaviour of grains during uniaxial compression at a specific orientation while the indentation modulus represents the average elastic response of differently oriented grains under a multiaxial stress field. The analysis of the elastic properties of the micropillars revealed that the measured Young’s modulus values are in good agreement with the forecasts of the DFT simulations for the monocarbides, suggesting their accurate prediction for grains of various crystallographic orientations. Presumably, this method also works for high-entropy carbides, which requires further DFT simulations to test this.

**Table 1:** Micromechanical properties of mono- and high-entropy carbides.

Material	Young’s modulus, *E* (GPa)	Young’s modulus, *E*_calc_ (GPa)	Yield stress, *σ*_y_ (GPa)	Critical resolved shear stress, *τ*_CRSS_ (GPa)	Nanohardness, *H* (GPa)*
HfC	458 ± 9	485 ± 5	3.93 ± 0.28	1.77 ± 0.13	31.5 ± 1.3
TaC	563 ± 34	565 ± 9	3.03 ± 0.11	1.36 ± 0.05	20.6 ± 1.2
(Hf-Ta-Zr-Nb)C	551 ± 24	n.a.	6.20 ± 0.12	2.79 ± 0.05	36.1 ± 1.6

Measured Young’s modulus, yield stress and calculated Young’s modulus, critical resolved shear stress for $\{110\}\langle 1\bar{1}0\rangle$ slip systems for HfC, TaC and (Hf-Ta-Zr-Nb)C micropillars, together with the nanohardness of the tested samples. *Castle *et al*.^11^.

The plastic behaviour of the micropillars was tested under uniaxial stress conditions in a specific orientation. As mentioned, micropillars were fabricated from grains with orientation of Φ ~ 14° and φ_2_ ~ 45°, which is rationalised as follows. We were interested both in the operation of slip systems in grains of polycrystalline mono- and high-entropy carbides and their critical resolved shear stress (CRSS) during uniaxial loading. In brief, the resolved shear stress (*τ*) measures the stress that acts in a given slip system exposed to a uniaxial stress field (*σ*), taking into account their relative orientation by the so-called Schmid factor (*m*) according to the below relationship.

2 $$\tau =\sigma \cdot m$$

**Figure 6:**
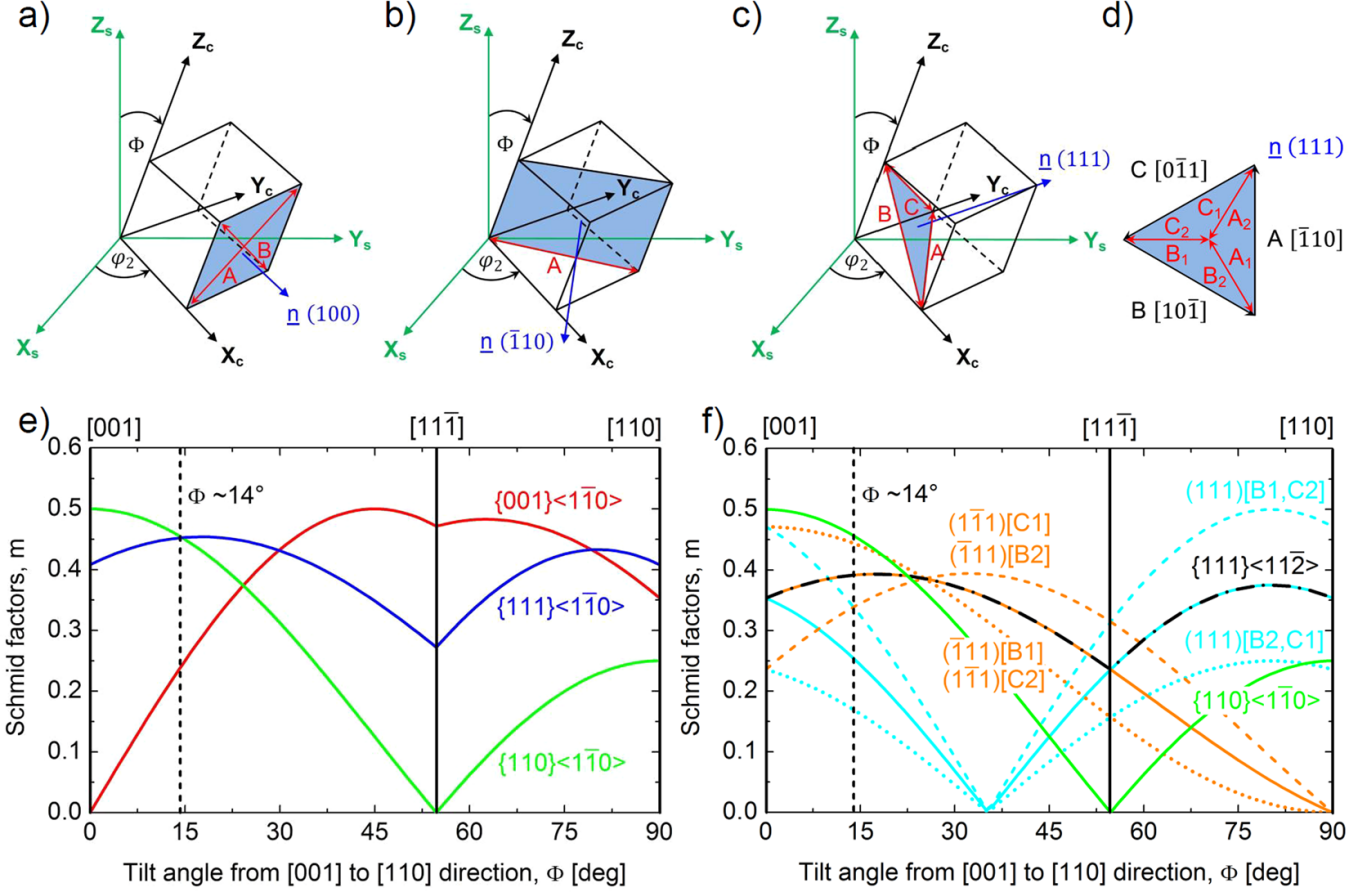
Slip systems and corresponding Schmid factors for mono- and high-entropy carbides. Schematic of operating slip planes and slip directions in (**a**) $\{001\}\langle 1\bar{1}0\rangle$, (**b**) $\{110\}\langle 1\bar{1}0\rangle$ and (**c**) $\{111\}\langle 1\bar{1}0\rangle$ slip systems. (**d**) The notation of $\frac{a}{6}\langle 112\rangle$ partial dislocations on {111} planes. (**e**) The orientation dependence of the maximal Schmid factors for $\{001\}\langle 1\bar{1}0\rangle$, $\{110\}\langle 1\bar{1}0\rangle$ and $\{110\}\langle 1\bar{1}0\rangle$ slip systems from [001] to [110] directions. (**f**) Orientation dependence of Schmid factors for the highest eight $\frac{a}{6}\langle 112\rangle$ type partial dislocations that can accomplish four different slips in the $\{111\}\langle 1\bar{1}0\rangle$ slip systems – (111)[*B*], (111)[*C*], $(\bar{1}11)[B]$, $(1\bar{1}1)[C]$ – and their average as the maximum for the $\{111\}\langle 11\bar{2}\rangle$ systems.

The critical resolved shear stress (*τ*_*CRSS*_) is the critical stress value necessary to activate slip by nucleating or activating dislocations in that specific slip system. This was motivated by the work of Kiani *et al*. who did micro-compression on oriented TaC and ZrC single crystals along the [100], [110] and [111] directions^20^. It was reported that the CRSS for TaC are almost the same for the $\{110\}\langle 1\bar{1}0\rangle$ and $\{111\}\langle 1\bar{1}0\rangle$ slip systems. Carrying out the calculation using the Schmid factors and compressive stresses reported along the [100] and [110] directions, ${\tau }_{\{110\}\langle 1\bar{1}0\rangle }$ = 4–5 GPa and ${\tau }_{\{111\}\langle 1\bar{1}0\rangle }$ = 4.1–4.9 GPa CRSS values were obtained for the activation of $\{110\}\langle 1\bar{1}0\rangle$ and $\{111\}\langle 1\bar{1}0\rangle$ slip systems, respectively^20^. These results for TaC are inconsistent with the work of Yu *et al*. who predicted 30% lower theoretical CRSS values for the $\{110\}\langle 1\bar{1}0\rangle$ compared to the $\{111\}\langle 1\bar{1}0\rangle$ slip systems in the case of group IV and V TMCs^18^. Here, it is important to emphasize that dislocation nucleation processes have considerable influence on CRSS at the micro/nano scale, resulting in more than three times higher experimental values for homogeneous nucleation during nanoindentation than for the heterogeneous case under micropillar compression; as was reported by Bei *et al*.^29^. We are aware that the quantitative comparison of micropillar results with theoretical simulations, which use homogeneous dislocation nucleation, is not reasonable; but qualitatively, both of them should show a similar trend of critical resolved shear stresses. To analyse this contradiction and to study the effect of anisotropy on slip operation, Schmid factors were calculated for $\{001\}\langle 1\bar{1}0\rangle$, $\{110\}\langle 1\bar{1}0\rangle$ and $\{111\}\langle 1\bar{1}0\rangle$ type slip system families as a function of crystallographic orientation as shown schematically in Fig. 6a–c. The maximum of the Schmid factors corresponding to the $\{001\}\langle 1\bar{1}0\rangle$, $\{110\}\langle 1\bar{1}0\rangle$ and $\{111\}\langle 1\bar{1}0\rangle$ slip system families exhibited different orientation dependences from [001], through $[11\bar{1}]$ to [110] loading directions as shown in Fig. 6e. It was revealed that there is an intersection point for $\{110\}\langle 1\bar{1}0\rangle$ and $\{111\}\langle 1\bar{1}0\rangle$ families at the orientation of Φ ~ 14° and φ_2_ ~ 45° with a corresponding Schmid factor of m = 0.45. Thus, micropillars were fabricated from both mono- and high-entropy carbide grains at that specific orientation to be able to determine from one set of measurements which of the $\{110\}\langle 1\bar{1}0\rangle$ and $\{111\}\langle 1\bar{1}0\rangle$ has the lower CRSS. It is important to note that φ_2_ has only a negligible effect on the intersection point; the difference is less than ΔΦ = 2° at φ_2_ = 0°. Taking into account the results of the slip analyses of the micropillars mentioned above, it is concluded that the $\{110\}\langle 1\bar{1}0\rangle$ slip systems have lower critical resolved shear stress than that of the $\{111\}\langle 1\bar{1}0\rangle$ family with corresponding values of *τ*_*TaC*_ = 1.36 ± 0.05 GPa, *τ*_*HfC*_ = 1.77 ± 0.13 GPa and *τ*_*HEC*_ = 2.79 ± 0.05 GPa as listed in Table 1 (HEC denotes (Hf-Ta-Zr-Nb)C high-entropy carbide).

To resolve the contradiction between the works of Yu *et al*.^18^ and Kiani *et al*.^20^ and to understand the basic deformation processes in grains of mono- and high-entropy carbides, slip was investigated via the activation of $\frac{a}{6}\langle 112\rangle$ partial dislocationson {111} planes^17,18^ (see Fig. 6d). This requires about a 25% lower CRSS than that necessary for the $\{111\}\langle 1\bar{1}0\rangle$ slip systems, according to Yu *et al*.^18^. The orientation dependence of Schmid factors for $\frac{a}{6}\langle 112\rangle$ partials on the {111} planes were calculated at an orientation of φ_2_ = 45° from Φ = 0°–90° similar to that which was done for the $\{001\}\langle 1\bar{1}0\rangle$, $\{110\}\langle 1\bar{1}0\rangle$ and $\{111\}\langle 1\bar{1}0\rangle$ slip system families. Taking into account that each <110> slip direction (denoted by A, B and C in Fig. 6d) splits into two directions (A1, A2, B2, B2 and C1, C2 in Fig. 6d) and that their consecutive movements accomplish <110> type slip, the corresponding joint Schmid factors are assumed to be the average along each direction instead of the minimum or maximum of a pair. The maximum of these averaged $\{111\}\langle 11\bar{2}\rangle$ type systems, which accomplishes four different slip systems in the $\{111\}\langle 1\bar{1}0\rangle$ slip systems, are shown in Fig. 6f. When loading the crystal along the [110] direction (Φ = 90°), this plot predicts that only two $\{111\}\langle 1\bar{1}0\rangle$ slip systems could operate via the dissociation of $\frac{a}{2}[10\bar{1}]$ and $\frac{a}{2}[0\bar{1}1]$ dislocations to B1, B2 and C1, C2 partials with corresponding average Schmid factors of m = 0.35. Along the [001] crystal direction (Φ = 0°), the activation of partial dislocations in the $\{111\}\langle 11\bar{2}\rangle$ system could accomplish four $\{111\}\langle 1\bar{1}0\rangle$ slip systems, namely the $(111)[10\bar{1}]$, $(111)[0\bar{1}1]$, $(\bar{1}11)[10\bar{1}]$ and $(1\bar{1}1)[0\bar{1}1]$, with corresponding Schmid factors of m = 0.35. Taking into account that the CRSS of $\{111\}\langle 11\bar{2}\rangle$ are fairly similar to the $\{110\}\langle 1\bar{1}0\rangle$ slip systems for TaC^18^, the one which has the higher Schmid factor will be the one that operates. The analysis of Fig. 6f reveals that the $\{110\}\langle 1\bar{1}0\rangle$ family can operate at Φ = 0° and Φ = 14° against the $\{111\}\langle 11\bar{2}\rangle$ while at Φ = 90° the activation of $\{111\}\langle 11\bar{2}\rangle$ partial systems is favourable against the $\{110\}\langle 1\bar{1}0\rangle$ slip systems.

The proposed Schmid factor analysis of slip systems reveals a scenario for slip of transition metal monocarbides and high-entropy carbides under uniaxial stress conditions which is in agreement with both the experimental results of Kiani *et al*.^20^ and theoretical predictions of Yu *et al*.^18^, moreover, it explains the slip systems observed in the present work. In the case of group IV TMCs, where the dissociation of <110> dislocations is energetically not favourable^17,18^, slip takes place on the $\{110\}\langle 1\bar{1}0\rangle$ slip systems against $\{111\}\langle 1\bar{1}0\rangle$ for orientations where the Schmid factor of the former exceeds about 70% of the latter one (${m}_{\{110\}\langle 1\bar{1}0\rangle } > 0.7\,{m}_{\{111\}\langle 1\bar{1}0\rangle }$), since the CRSS of $\{110\}\langle 1\bar{1}0\rangle$ slip systems is about 30% lower than that of $\{111\}\langle 1\bar{1}0\rangle$ for TiC, ZrC and HfC^18^. This includes the orientations of Φ = 0° and Φ = 14° for ZrC by Kiani *et al*.^20^ and for HfC in the present work (see Fig. 6e), respectively, where the $\{110\}\langle 1\bar{1}0\rangle$ slip systems were reported. In the case of group V TMCs (e.g. TaC), where $\frac{a}{6}\langle 112\rangle$ partial dislocations can form on {111} planes, slip occurs directly on the $\{111\}\langle 1\bar{1}0\rangle$ slip systems against $\{110\}\langle 1\bar{1}0\rangle$ for orientations where the Schmid factor of the former exceeds about 137–157% of the latter one (${m}_{\{111\}\langle 1\bar{1}0\rangle } > 1.5\,{m}_{\{110\}\langle 1\bar{1}0\rangle }$), since the CRSS of $\{111\}\langle 1\bar{1}0\rangle$ slip systems is about 37–57% higher than that of $\{110\}\langle 1\bar{1}0\rangle$ for VC, NbC and TaC^18^. This condition is satisfied for Φ = 90° as ${m}_{\{111\}\langle 1\bar{1}0\rangle }/{m}_{\{110\}\langle 1\bar{1}0\rangle }=1.6$ (see Fig. 6e), allowing the operation of the $\{111\}\langle 1\bar{1}0\rangle$ slip systems. Assuming the direct activation of $\{111\}\langle 1\bar{1}0\rangle$ systems (without the aid of partial dislocations), it results in a similar CRSS for $\{110\}\langle 1\bar{1}0\rangle$ and $\{111\}\langle 1\bar{1}0\rangle$ slip systems^20^, which is in contradiction with the theoretical predictions^18^, and consequently does not explain the operation of $\{110\}\langle 1\bar{1}0\rangle$ against $\{111\}\langle 1\bar{1}0\rangle$ slip systems at Φ = 14° obtained in the present workbased on their Schmid factors (see Fig. 6e). All of this suggests that the activation of <112> dislocations on {111} planes is plausible. This occurs for orientations where the Schmid factor of $\{111\}\langle 11\bar{2}\rangle$ systems exceeds about 103–119% of the values of $\{110\}\langle 1\bar{1}0\rangle$ slip systems (${m}_{\{111\}\langle 11\bar{2}\rangle } > 1.19\,{m}_{\{110\}\langle 1\bar{1}0\rangle }$), since the CRSS of the former is predicted to be about 3–19% higher than that of the latter one for VC, NbC and TaC^18^. Considering TaC, which has only 3% higher CRSS, this condition is fulfilled at Φ = 90° in agreement with the results of Kiani *et al*.^20^ but fails at Φ = 14° (see Fig. 6f), confirming the operation of the $\{110\}\langle 1\bar{1}0\rangle$ slip systems found in the present work.

Here it is important to emphasize that the slip activation of TMC grains outlined above is confined to uniaxial stress condition at a specific orientation which could result in the operation of $\{110\}\langle 1\bar{1}0\rangle$ slip systems. This is consistent with the expectation for group IV TMCs crystals in which the $\{110\}\langle 1\bar{1}0\rangle$ slip systems were found to be energetically favourable in various experiments^14–17^. However, it differs that reported for group V TMCs, where the $\{111\}\langle 1\bar{1}0\rangle$ slip systems were observed to be the dominant during indentation tests^14–17^. This paradox is attributed to the different Schmid factors in each case due to their different stress fields (uniaxial for micro-compression and multiaxial for indentation)^30^ and the effect of grain orientation. Since the determination of Schmid factors for indentation is difficult^30^, our nanohardness results^11^ cannot be compared directly with CRSS values listed in Table 1 but qualitative predictions can made for the dominant slip systems during nanoindentation. Here, it is important to note that our nanoindentation work^11^ was also carried out in randomly oriented grains of the same sample that were used for micropillar compression. Assuming that the dominant slip systems are the $\{110\}\langle 1\bar{1}0\rangle$ type both in HfC and TaC, the hardness ratio should be similar to the CRSS ratio since the corresponding Schmid factors are the same during indentation. Note that CRSS values may differ for indentation and micro-compression due to different dislocation nucleation (homogeneous vs. heterogeneous)^29^ but their ratio are assumed to be equal. The ratio of CRSS values for HfC and TaC is *τ*_*HfC*_/*τ*_*TaC*_ = 1.30 for $\{110\}\langle 1\bar{1}0\rangle$ slip systems while their hardness ratio is *H*_*HfC*_/*H*_*TaC*_ = 1.53 which suggests different dominant slip systems during indentation in agreement with the literature^14–17^. The higher hardness ratio fits well with the operation of $\{111\}\langle 1\bar{1}0\rangle$ observed in TaC which results in lower hardness (therefore higher *H*_*HfC*_/*H*_*TaC*_) compared to the case of the activation of $\{110\}\langle 1\bar{1}0\rangle$ systems found in substoichiometric compositions of group V monocarbides^15,16,27^. Using the similar comparison of CRSS and hardness ratios for HfC and (Hf-Ta-Zr-Nb)C, the dominant slip system of (Hf-Ta-Zr-Nb)C is predicted during indentation as follows. Assuming that the dominant slip systems are the $\{110\}\langle 1\bar{1}0\rangle$ type in both carbides, their CRSS and hardness ratios expected to be equal similar to that was explained before. Based on Table 1, the ratio of CRSS values for (Hf-Ta-Zr-Nb)C (denoted as HEC) and HfC is obtained to *τ*_*HEC*_/*τ*_*HfC*_ = 1.58 while their hardness ratio is *H*_*HEC*_/*H*_*HfC*_ = 1.15 which suggests different dominant slip systems during indentation that the $\{110\}\langle 1\bar{1}0\rangle$ identified in micropillar compression. Since the dominant slip systems in TaC and NbC is the $\{111\}\langle 1\bar{1}0\rangle$^16,17^ and this slip systems was observed also in binary (Hf-Ta)C during indentation^19^, it suggests that the operation of $\{111\}\langle 1\bar{1}0\rangle$ slip systems is likely to be the dominant during nanoindentation of high-entropy carbide. The dominance of $\{111\}\langle 1\bar{1}0\rangle$ against $\{110\}\langle 1\bar{1}0\rangle$ systems, which results more ductility and reduced hardness, is in agreement with the lower nanohardness enhancement of (Hf-Ta-Zr-Nb)C compared to HfC than it is expected based on their CRSS ratio during micro-compression.

In conclusion, the much larger strength enhancement of micropillars measured in the present work compared to the average nanohardness of randomly oriented grains is attributed to the different slip systems. In the present work for (Hf-Ta-Zr-Nb)C, the operation of $\{110\}\langle 1\bar{1}0\rangle$ was identified in micropillar experiments, but the dominant slip system in nanoindentation is assumed to be the $\{111\}\langle 1\bar{1}0\rangle$, possibly via the activation of partial dislocations, which is attributed to the different Schmid factors due to the different stress fields between nanoindentation and micropillar compression.

## Methods

### Material preparation and characterization

Bulk, single phase polycrystalline HfC, TaC monocarbides and (Hf-Ta-Zr-Nb)C high-entropy carbide samples were synthesized from carefully selected, high purity raw powders (HfC and TaC from H.C. Starck, ZrC and NbC from American Elements) using ball milling and spark plasma sintering (SPS). The carbide powders were weighed in equiatomic proportions and mixed at 200 rpm for 24 hr in WC pots. Then the powder mixture was compacted into a 20 mm diameter graphite die and SPS processed (FCT HPD 25, FCT Systeme GmbH) applying a two-step optimised heating profile with a 10 min dwell at 1800 °C and a 2 min dwell at 2300 °C in a vacuum of ~5 Pa. The loading pressure was increased from the minimum of 16 MPa to 40 MPa during the dwell at 1800 °C and was reduced back down to 16 MPa at 2300 °C. More details of powder characterization, selection and SPS processing can be found in our recent paper^11^.

Prior to micropillar compression, the prepared materials (HfC, TaC and (Hf-Ta-Zr-Nb)C were subjected to standard metallographic procedures (cutting, grinding, polishing) to prepare an appropriate surface for scanning electron microscopy (SEM) observation and electron backscatter diffraction (EBSD) analysis on a FEI Quanta 3D machine. The crystallographic orientations of mono- and high-entropy transition metal carbide grains (rock salt crystal structure, space group Fm-3m, No. 225) were determined on the basis of the measured EBSD map using Orientation Imaging (OIM) software (EDAX). The program characterizes the crystal orientations in terms of the Euler angles (${\phi }_{1},{\rm{\Phi }},{\phi }_{2}$) that are needed to bring the principal axes of the sample (X_S_, Y_S_, Z_S_) into coincidence with the crystal coordinate system (X_C_, Y_C_, Z_C_). Due to the rotational symmetry of micro-compression, the only relevant Euler angles are Φ and *φ*_2_ as the sample can always be rotated by *φ*_1_ around Z_S_ to bring X_S_ into coincidence with the line of intersecting planes (see line in Supplementary Fig. 4). In the present work, crystals were investigated at *φ*_2_ ~ 45°, which resulted in Φ representing their tilt angle from the [001] to [110] direction.

From the obtained samples (HfC, TaC and (Hf-Ta-Zr-Nb)C), large grains with orientations of Φ ~ 14° at *φ*_2_ ~ 45° and misorientations of ${\rm{\Delta }}{\rm{\Phi }} < 2^\circ$ and ${\rm{\Delta }}{\phi }_{2} < 21^\circ$ were selected for micropillar fabrication using the focused ion beam (FIB) technique (FEI Quanta 3D), as shown in Fig. 1d. The selection of the above defined orientations is rationalized in the section on ‘Calculation of Schmid-factors’. In each sample, four micropillars with a diameter of ~2.5 μm, a height of ~5 μm and a trench diameter around them of ~15 μm were milled out of pore-free regions of the selected grains.

### Micropillar compression

Micropillar compressions were carried out at room temperature on an Agilent/Keysight G200 nanoindenter equipped with a flat-punch diamond indenter tip of diameter of 5 μm. The machine operates in load-controlled mode with the possibility of an instantaneous stop and unload option to avoid the further damaging effect after any interesting point (e.g. elastic limit) on the loading curve. The maximum load was adjusted to 100 mN, the loading/unloading rate was 0.02 mN/s with a dwell time of 10 s between loading and unloading. Each measurement was corrected for drift, which was about 0.1–0.2 nm/s. The compliance of the nanoindentation machine was calibrated prior to the measurements, and the accuracy of the load cell was certified by the producer. The compliance of the tip-micropillar-substrate material system was taken into consideration using the Sneddon model^31^. The measured raw displacement (*h*_*raw*_) data were corrected by the deflection of the diamond indenter tip (Δ*h*_i_) at the top of the pillars and the deflections of the substrate materials (Δ*h*_s_) at their bottom using the below equations where *r*_0_, *R*_0_ and *F* denote the initial top and bottom diameters of the micropillars and load, respectively.

3 $$h={h}_{raw}-{\rm{\Delta }}{h}_{i}-{\rm{\Delta }}{h}_{s}$$

4 $${\rm{\Delta }}{h}_{{\rm{i}}}=\frac{1-{\nu }_{i}^{2}}{{E}_{i}}\cdot \frac{F}{2{r}_{0}}$$

5 $${\rm{\Delta }}{h}_{{\rm{s}}}=\frac{1-{\nu }_{s}^{2}}{{E}_{s}}\cdot \frac{F}{2{R}_{0}}=\frac{1}{{M}_{s}}\cdot \frac{F}{2{R}_{0}}$$

The Young’s modulus and Poisson’s ratio of the indenter used were *E*_i_ = 1141 GPa and *ν*_i_ = 0.07, respectively. In the case of the substrate materials, the indentation modulus (*M*_s_) was used according to the $\frac{1-{\nu }_{s}^{2}}{{E}_{s}}=\frac{1}{{M}_{s}}$ equation. The corresponding values for HfC, TaC and (Hf-Ta-Zr-Nb)C were selected to *M*_HfC_ = 552 GPa, *M*_TaC_ = 579 GPa and *M*_HEC_ = 598 GPa respectively, according to our nanoindentation measurements^11^. The engineering stress ($\sigma =\frac{F}{{r}_{avg}^{2}\pi }$) and engineering strain ($\varepsilon =\frac{h}{{L}_{0}}$) were calculated using the measured load, corrected displacement, initial length (*L*_0_) of the micropillars and their average radius (*r*_*avg*_) due to tapering. All of the micropillars were examined in a scanning electron microscope (SEM) before and after the micro-compressions to study their deformation and to eliminate those that failed due to unwanted local defects (e.g. pores, pre-existing cracks), acting as stress enhancers. The Young’s modulus (*E*) and yield stress (*σ*_y_) of the micropillars were determined from the slope of unloading part of the engineering stress-strain curves, which behaves purely elastically according to Hooke’s law (*σ* = *Eε*), and the offset flow stress at 0.2% of strain, respectively. The averaged *E* and *σ*_y_ values are the average of at least 3 curves on each sample.

### Calculation of Young’s modulus

The Young’s modulus of a rotated crystal possessing cubic symmetry, like HfC, TaC and (Hf-Ta-Zr-Nb)C of rock salt crystal structure, can be derived from the rotation of the corresponding elastic stiffness tensor. For cubic symmetry, the contracted elastic stiffness tensor (*C*_ij_) consists of three elastic constants (*c*_11_, *c*_12_ and *c*_44_) in the following form^32^:

6 $${C}_{ij}=(\begin{array}{cccccc}{c}_{11} & {c}_{12} & {c}_{12} & 0 & 0 & 0\\ {c}_{12} & {c}_{11} & {c}_{12} & 0 & 0 & 0\\ {c}_{12} & {c}_{12} & {c}_{11} & 0 & 0 & 0\\ 0 & 0 & 0 & {c}_{44} & 0 & 0\\ 0 & 0 & 0 & 0 & {c}_{44} & 0\\ 0 & 0 & 0 & 0 & 0 & {c}_{44}\end{array})$$

The Young’s modulus values along the principal axes of a crystal (X_C_, Y_C_, Z_C_) along [100], [010] and [001] directions are the reciprocal of the corresponding components of the compliance tensor (*S*_ij_), which is the inverse of the stiffness tensor (*S*_*ij*_ = (*C*_*ij*_)^−1^), according to *E*_*x*_ = 1/*s*_11_, *E*_*y*_ = 1/*s*_22_ and *E*_*z*_ = 1/*s*_33_. In the case of a rotated crystal as shown in Fig. 6a–c, the Young’s modulus along the Z_S_ axis (loading direction of micropillars) can be determined as the reciprocal of (*s*_33_) component of the compliance tensor in the sample coordinate system as *E* = 1/(*s*_33_)′. To this, the crystal is subjected to a rotation of *φ*_2_ = 45° around Z_C_ and that is followed by an arbitrary rotation of Φ around X_S_. The counter clockwise rotation of *C*_ij_ around Z_C_ by an angle of *φ*_2_ a in the crystal coordinate system can be carried out by the transformation of ${T}_{Z}\cdot {C}_{ij}\cdot {T}_{Z}^{T}$, where *T*_*Z*_ represents the below rotation tensor and ${T}_{Z}^{T}$ is the transpose of it^32^.

7 $${T}_{Z}=(\begin{array}{cccccc}{\cos }^{2}{\phi }_{2} & {\sin }^{2}{\phi }_{2} & 0 & 0 & 0 & 2\,\sin \,{\phi }_{2}\,\cos \,{\phi }_{2}\\ {\sin }^{2}{\phi }_{2} & {\cos }^{2}{\phi }_{2} & 0 & 0 & 0 & -2\,\sin \,{\phi }_{2}\,\cos \,{\phi }_{2}\\ 0 & 0 & 1 & 0 & 0 & 0\\ 0 & 0 & 0 & \cos \,{\phi }_{2} & -\,\sin \,{\phi }_{2} & 0\\ 0 & 0 & 0 & \sin \,{\phi }_{2} & \cos \,{\phi }_{2} & 0\\ -\,\sin \,{\phi }_{2}\,\cos \,{\phi }_{2} & \sin \,{\phi }_{2}\,\cos \,{\phi }_{2} & 0 & 0 & 0 & {\cos }^{2}{\phi }_{2}-{\sin }^{2}{\phi }_{2}\end{array})$$

In the case of a rotation of *φ*_2_ = 45°, the stiffness tensor has the below form:

8 $${C}_{ij}^{45^\circ }=(\begin{array}{cccccc}\frac{{c}_{11}+{c}_{12}}{2}+{c}_{44} & \frac{{c}_{11}+{c}_{12}}{2}-{c}_{44} & {c}_{12} & 0 & 0 & 0\\ \frac{{c}_{11}+{c}_{12}}{2}-{c}_{44} & \frac{{c}_{11}+{c}_{12}}{2}+{c}_{44} & {c}_{12} & 0 & 0 & 0\\ {c}_{12} & {c}_{12} & {c}_{11} & 0 & 0 & 0\\ 0 & 0 & 0 & {c}_{44} & 0 & 0\\ 0 & 0 & 0 & 0 & {c}_{44} & 0\\ 0 & 0 & 0 & 0 & 0 & \frac{{c}_{11}-{c}_{12}}{2}\end{array})$$

For the determination of Young’s modulus as function of Φ tilt angle, the compliance tensor is calculated as the inverse of ${C}_{ij}^{45^\circ }$ according to ${S}_{ij}^{45^\circ }={({C}_{ij}^{45^\circ })}^{-1}$. This compliance tensor is subjected to clockwise rotation of Φ around X_S_ (see Fig. 6a–c) according to formula of $({S}_{ij}^{45^\circ })^{\prime} ={({T}_{X}^{-1})}^{T}\cdot {S}_{ij}^{45^\circ }\cdot {T}_{X}^{-1}$, where *T*_*X*_ is the below rotation tensor, ${T}_{X}^{-1}$ is its inverse and ${({T}_{X}^{-1})}^{T}$ is the transpose of it^32^.

9 $${T}_{X}=(\begin{array}{cccccc}1 & 0 & 0 & 0 & 0 & 0\\ 0 & {\cos }^{2}{\rm{\Phi }} & {\sin }^{2}{\rm{\Phi }} & -2\,\sin \,{\rm{\Phi }}\,\cos \,{\rm{\Phi }} & 0 & 0\\ 0 & {\sin }^{2}{\rm{\Phi }} & {\cos }^{2}{\rm{\Phi }} & 2\,\sin \,{\rm{\Phi }}\,\cos \,{\rm{\Phi }} & 0 & 0\\ 0 & \sin \,{\rm{\Phi }}\,\cos \,{\rm{\Phi }} & -\,\sin \,{\rm{\Phi }}\,\cos \,{\rm{\Phi }} & {\cos }^{2}{\rm{\Phi }}-\,{\sin }^{2}{\rm{\Phi }} & 0 & 0\\ 0 & 0 & 0 & 0 & \cos \,{\rm{\Phi }} & \sin \,{\rm{\Phi }}\\ 0 & 0 & 0 & 0 & -\,\sin \,{\rm{\Phi }} & \cos \,{\rm{\Phi }}\end{array})$$

The Young’s modulus, as the reciprocal of $({s}_{33}^{45^\circ })^{\prime}$ component, is derived as follows:

10 $$E=1/({s}_{33}^{45^\circ })^{\prime} ={({s}_{22}^{45^\circ }{\sin }^{4}{\rm{\Phi }}+(2{s}_{23}^{45^\circ }+{s}_{44}^{45^\circ }){\sin }^{2}{{\rm{\Phi }}\cos }^{2}{\rm{\Phi }}+{s}_{33}^{45^\circ }{\cos }^{4}{\rm{\Phi }})}^{-1}$$

where the ${s}_{22}^{45^\circ }$, ${s}_{23}^{45^\circ }$, ${s}_{33}^{45^\circ }$ and ${s}_{44}^{45^\circ }$ are the components of ${S}_{ij}^{45^\circ }={({C}_{ij}^{45^\circ })}^{-1}$ tensor derived from the elastic constants using Eq. (8).

The elastic constants of cubic crystals used were *c*_11_ = 540, *c*_12_ = 112, *c*_44_ = 171 and *c*_11_ = 674, *c*_12_ = 172, *c*_44_ = 167 in GPa for HfC and TaC, respectively^19^. Calculations were carried out according to Eq. (10) for crystallographic orientations covering the [001], $[11\bar{1}]$ and [110] directions by rotating the crystal from Φ = 0° to Φ = 90° at φ_2_ = 45° as shown schematically in Fig. 6a–c. The scatter of the calculated data corresponds to a ΔΦ = 2° misorientation angle around the Φ = 14° at φ_2_ = 45° orientations, in agreement with the experiments. It is important to note that the variation of φ_2_ from 0° to 45° has a negligible effect on *E*_calc_.

### Calculation of Schmid factors

In order to analyse the anisotropic slip activation and to determine the most favourable slip systems for an oriented rocksalt (B1) crystal structure, Schmid factors were calculated for the $\{001\}\langle 1\bar{1}0\rangle$, $\{110\}\langle 1\bar{1}0\rangle$ and $\{111\}\langle 1\bar{1}0\rangle$ type slip system families as a function of crystallographic orientation. The selection of these slip systems was based on the assumption that transition metal monocarbides can undergo plastic deformation via slip on all three low-index planes – {100}, {110} and {111} – along the <110> directions^15–17,27^, and similar behaviour was expected for the (Hf-Ta-Zr-Nb)C high-entropy carbide prior to the micropillar compression testing. Slip systems, as a given plane and a line on it, were defined in the coordinate system of the crystal (X_C_, Y_C_, Z_C_) as shown in Fig. 6. There are six $\{001\}\langle 1\bar{1}0\rangle$ type slip systems which consist of three different slip planes (one normal for each opposite cubic faces corresponding to (100), (010) and (001) planes) and the corresponding two different slip directions along the face diagonals (denoted by A and B). The $\{110\}\langle 1\bar{1}0\rangle$ family contains six slip systems, building from six different planes (one for each opposite cubic edges corresponding to the (110), $(\bar{1}10)$, $(10\bar{1})$, (101), (011) and $(01\bar{1})$ planes) and one slip directions for each (A). The $\{111\}\langle 1\bar{1}0\rangle$ family consists of twelve slip systems based on four different slip planes (one normal for each opposite cubic vertex corresponding to the (111), $(\bar{1}11)$, $(\bar{1}\bar{1}1)$ and $(1\bar{1}1)$ planes) and the corresponding three slip directions (A, B and C). Slip plane normal (${\underline{n}}_{(hkl)}$) and slip direction (${\underline{v}}_{(hkl)i},i=A,B,C$) vectors for slip systems shown in Fig. 6 are as follows:

11 $${\underline{n}}_{(100)}=(\begin{array}{c}1\\ 0\\ 0\end{array})\,with\,{\underline{v}}_{(100)A}=(\begin{array}{c}0\\ 1\\ 1\end{array})/\sqrt{2}\,and\,{\underline{v}}_{(100)B}=(\begin{array}{c}0\\ 1\\ -1\end{array})/\sqrt{2}$$

12 $${\underline{n}}_{(\bar{1}10)}=(\begin{array}{c}-1\\ 1\\ 0\end{array})/\sqrt{2}\,with\,{\underline{v}}_{(\bar{1}10)A}=(\begin{array}{c}1\\ 1\\ 0\end{array})/\sqrt{2}$$

13 $${\underline{n}}_{(111)}=(\begin{array}{c}1\\ 1\\ 1\end{array})/\sqrt{3}\,with\,{\underline{v}}_{(111)A}=(\begin{array}{c}-1\\ 1\\ 0\end{array})/\sqrt{2},\,{\underline{v}}_{(111)B}=(\begin{array}{c}-1\\ 0\\ 1\end{array})/\sqrt{2}\,and\,{\underline{v}}_{(111)C}=(\begin{array}{c}0\\ -1\\ 1\end{array})/\sqrt{2}$$

The rest of the slip systems were calculated by their consecutive rotations of 90° around X_C_, Y_C_ and Z_C_ (see Supplementary data).

In addition to the above defined slip systems, partial slips were also considered on the {111} planes with slip directions of <112> type as shown in Fig. 6d. The $\{111\}\langle 11\bar{2}\rangle$ type systems consist of twenty-four partial slip systems based on the four {111} type planes and the corresponding six <112> directions of which two consecutive directions (denoted as A_1_, A_2_, B_1_, B_2_ and C_1_, C_2_) form a <110> type slip. Coordinates of slip directions on the (111) plane are defined as follows:

14 $${\underline{v}}_{(111)A1}=(\begin{array}{c}-1\\ \frac{1}{2}\\ \frac{1}{2}\end{array})/\sqrt{\frac{6}{4}},\,{\underline{v}}_{(111)A2}=(\begin{array}{c}-\frac{1}{2}\\ 1\\ -\frac{1}{2}\end{array})/\sqrt{\frac{6}{4}},\,{\underline{v}}_{(111)B1}=(\begin{array}{c}\frac{1}{2}\\ \frac{1}{2}\\ -1\end{array})/\sqrt{\frac{6}{4}}$$

15 $${\underline{v}}_{(111)B2}=(\begin{array}{c}1\\ -\frac{1}{2}\\ -\frac{1}{2}\end{array})/\sqrt{\frac{6}{4}},\,{\underline{v}}_{(111)C1}=(\begin{array}{c}\frac{1}{2}\\ -1\\ \frac{1}{2}\end{array})/\sqrt{\frac{6}{4}},\,{\underline{v}}_{(111)C2}=(\begin{array}{c}-\frac{1}{2}\\ -\frac{1}{2}\\ 1\end{array})/\sqrt{\frac{6}{4}}$$

The rest of the slip systems corresponding to the other {111} type planes were calculated by their consecutive rotations of 90° around Z_C_ as was done for the $\{111\}\langle 1\bar{1}0\rangle$ slip systems.

In order to determine Schmid factors for the above listed slip systems covering the <100>, <110> and <111> crystallographic directions, slip plane normal and slip direction vectors those were subjected to rotations around Z_S_ by *φ*_2_ = 45° and subsequently around X_S_ by an arbitrary Φ angle from 0° to 90°in the sample coordinate systems (X_S_, Y_S_, Z_S_) as shown in Fig. 6. The [001], $[11\bar{1}]$ and [110] directions correspond to ${\rm{\Phi }}=0^\circ$, ${\rm{\Phi }}=54.7^\circ$ and ${\rm{\Phi }}=90^\circ$ rotations at *φ*_2_ = 45°, respectively.The rotation of slip planes and directions were calculated as follows:

16 $${\underline{n}}_{ROT}({\rm{\Phi }})={\hat{R}}_{X}\cdot ({\hat{R}}_{Z}\cdot \underline{n})\,{\rm{and}}\,{\underline{\nu }}_{ROT}({\rm{\Phi }})={\hat{R}}_{X}\cdot ({\hat{R}}_{Z}\cdot \underline{\nu })$$

The ${\hat{R}}_{Z}$ and ${\hat{R}}_{X}$ represent positive (counter clockwise) and negative (clockwise) rotations around Z_S_ and X_S_, respectively (see Fig. 6).

17 $${\hat{R}}_{Z}=(\begin{array}{ccc}{\cos {\rm{\phi }}}_{{\rm{2}}} & -\,{\sin {\rm{\phi }}}_{{\rm{2}}} & {\rm{0}}\\ {\sin {\rm{\phi }}}_{{\rm{2}}} & {\cos {\rm{\phi }}}_{{\rm{2}}} & {\rm{0}}\\ {\rm{0}} & {\rm{0}} & {\rm{1}}\end{array})\,{\rm{and}}\,{\hat{R}}_{X}=(\begin{array}{ccc}{\rm{1}} & {\rm{0}} & {\rm{0}}\\ {\rm{0}} & \cos \,{\rm{\Phi }} & \sin \,{\rm{\Phi }}\\ {\rm{0}} & -\,\sin \,{\rm{\Phi }} & \cos \,{\rm{\Phi }}\end{array})$$

The Schmid-factors (*m*) were calculated for a given slip system as follows:

18 $$m=\,\cos \,\alpha \cdot \,\cos \,\beta =|F\cdot {\underline{n}}_{ROT}({\rm{\Phi }})|\cdot |F\cdot {\underline{\nu }}_{ROT}({\rm{\Phi }})|$$

where the *F* denotes the normalized load as $$F=(\begin{array}{c}0\\ 0\\ -1\end{array})$$, ${\underline{n}}_{ROT}({\rm{\Phi }})$ and ${\underline{\nu }}_{ROT}({\rm{\Phi }})$ represent the slip plane normal and slip direction for a rotated crystal in the sample coordinate system (X_S_, Y_S_, Z_S_). The absolute values were applied in the expressions of cos*α* and cos*β* to maintain that the *α* and *β* values be lower than 90°. This means that the slip plane normal is selected so that $\underline{F}\cdot \underline{n} > 0$ and the relevant slip direction is selected from a specific slip line (A, B or C) which satisfies the $\underline{F}\cdot \underline{\nu } > 0$ condition.

## Supplementary information

Dataset 1

## Data Availability

The data that support the findings of this study are available from the corresponding author on reasonable request.
