# Supplementary material for: Strength enhancement and slip behaviour of high-entropy carbide grains during micro-compression
Source: Sci Rep. 2019 Jul 15;9:10200. doi: 10.1038/s41598-019-46614-w (PMC6629678; doi:10.1038/s41598-019-46614-w)
Supplement: Supplementary file 1 — Dataset 1 [file 41598_2019_46614_MOESM1_ESM.docx]

**Strength enhancement and slip behaviour of high-entropy carbide grains during micro-compression**

Tamás Csanádi^1,*^, Elinor Castle^2^, Michael J. Reece^2^ & Ján Dusza^1^

^1^Institute of Materials Research, Slovak Academy of Sciences, Watsonova 47, 04353 Košice, Slovak Republic.

^2^School of Engineering and Material Science, Queen Mary University of London, London, E1 4NS, UK. Correspondence and requests for materials should be addressed to T.Cs. (email: tcsanadi@saske.sk).

**Supplementary Information**


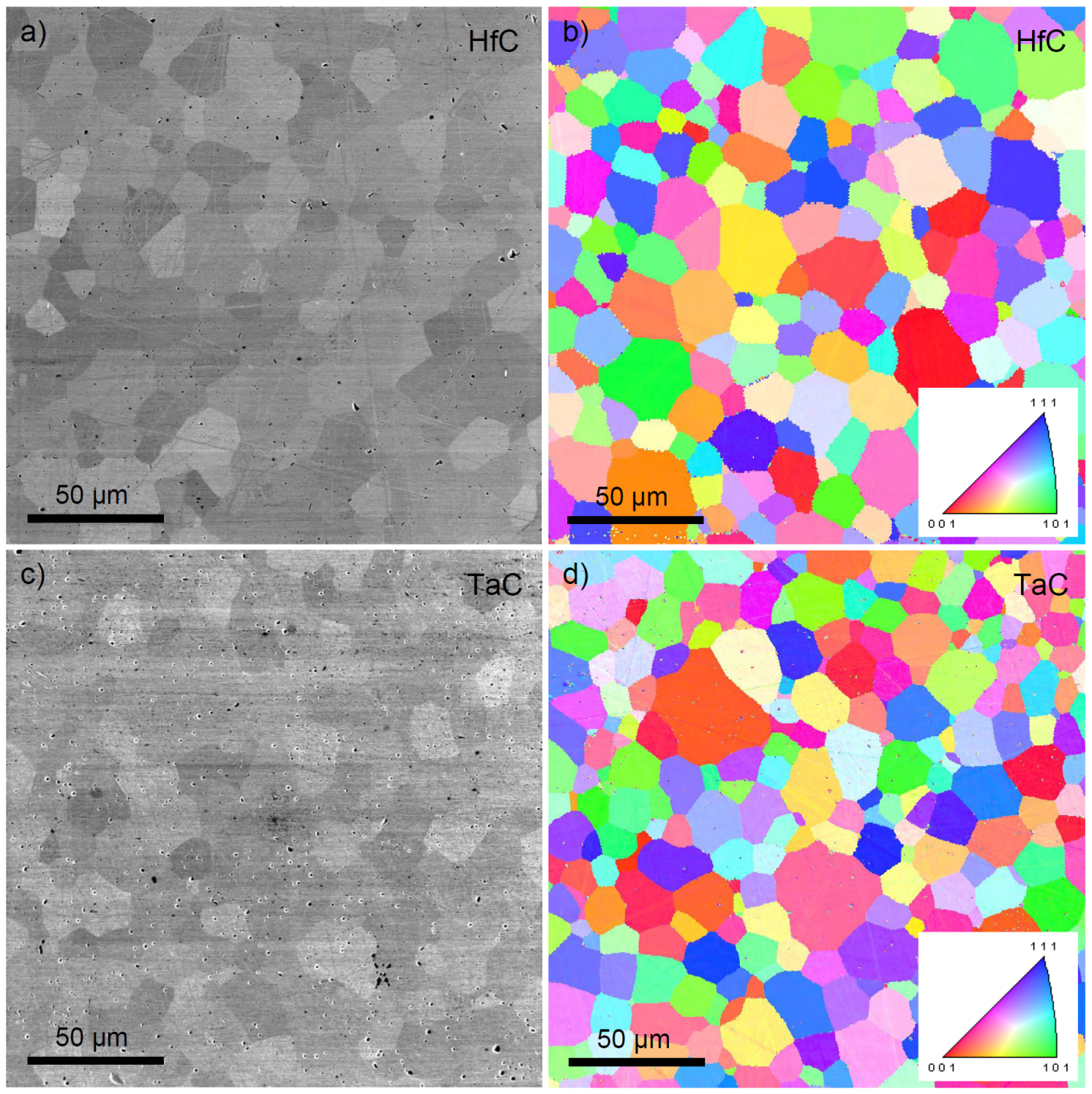


**Supplementary Figure 1:** Microstructure of polycrystalline bulk HfC and TaC samples by a), c) SEM and b), d) EBSD.

**
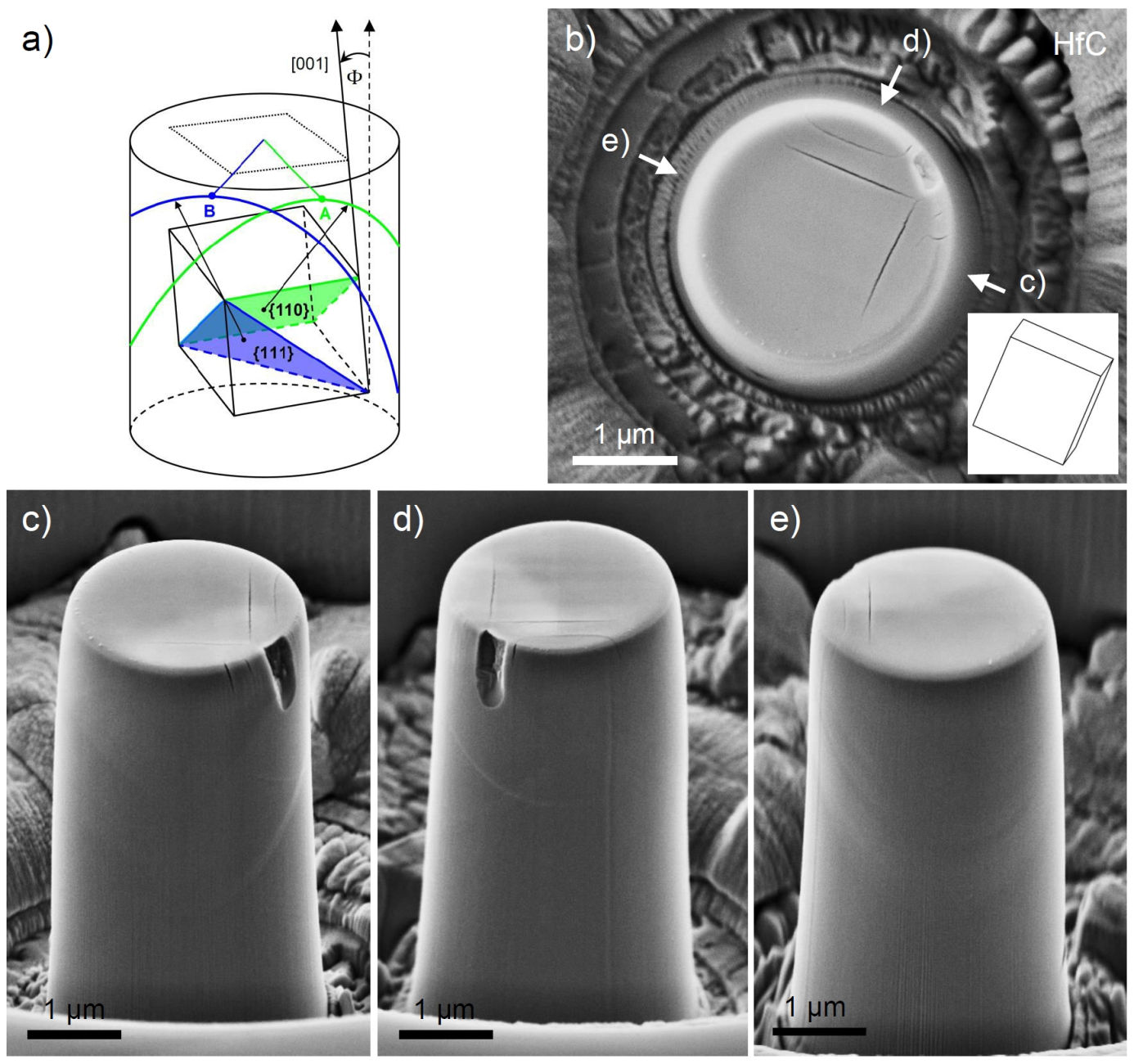
**

**Supplementary Figure 2:** a) Slip patterns expected to be formed on pillar surfaces during the operation of {110} and {111} type slip planes. b) Top view of a compressed HfC micropillar with the inset of the corresponding crystal orientation determined by EBSD. c)-e) Side view SEM images of the compressed micropillars rotated by 90° relative to each other as shown in Suppl. Fig. 2b.

**
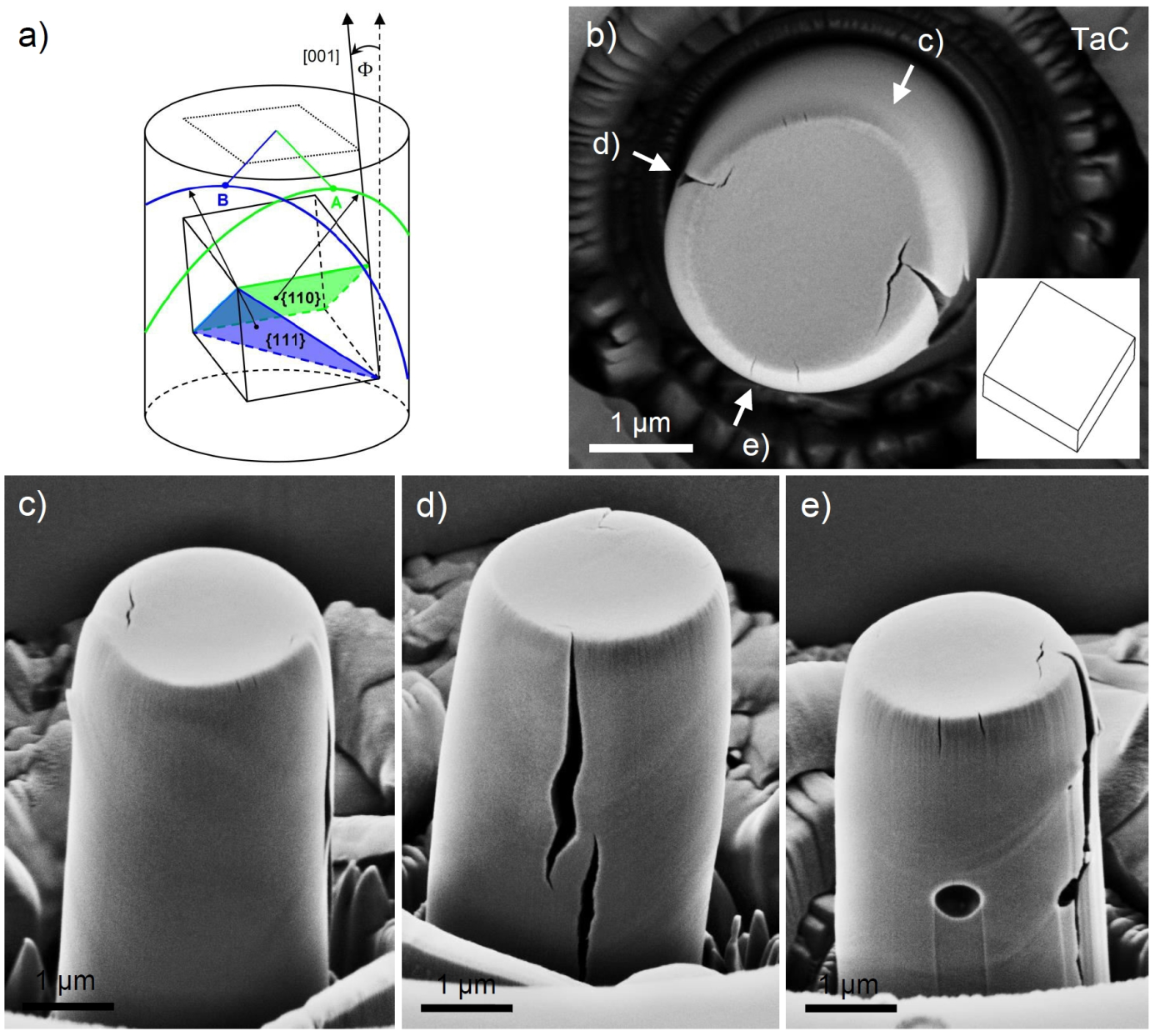
**

**Supplementary Figure 3:** a) Slip patterns expected to be formed on pillar surfaces during the operation of {110} and {111} type slip planes. b) Top view of a compressed TaC micropillar with the inset of the corresponding crystal orientation determined by EBSD. c)-e) Side view SEM images of the compressed micropillars rotated by 90° relative to each other as shown in Suppl. Fig. 3b.

**
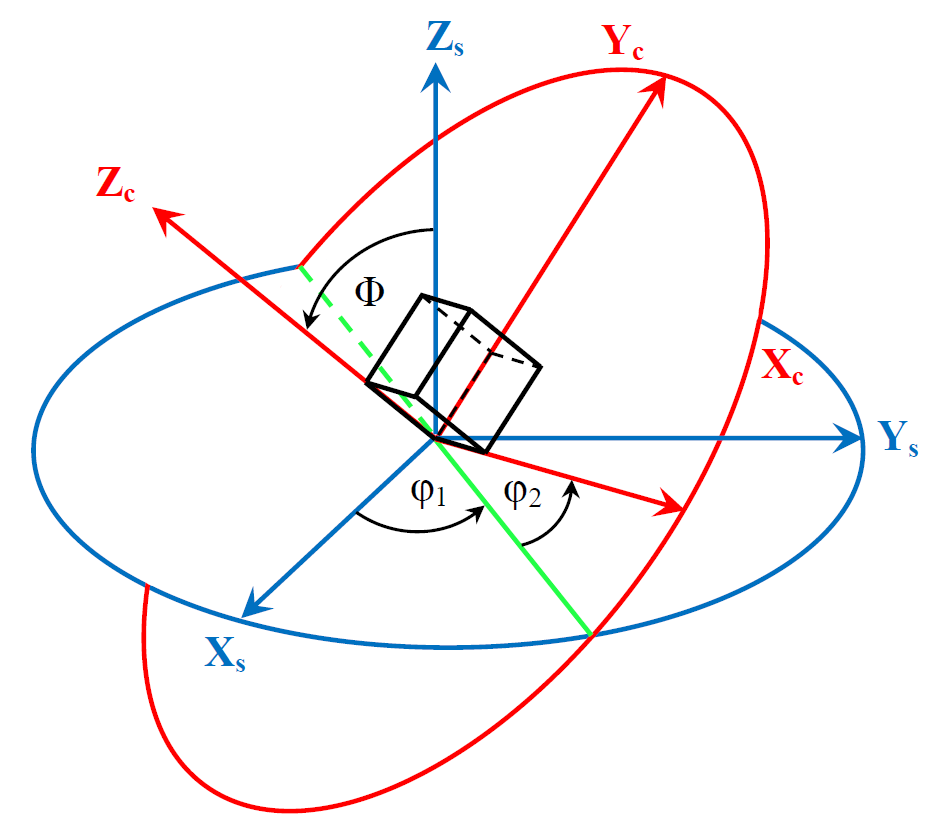
**

**Supplementary Figure 4:** Schematic of the orientation of principal axes of the crystal relative to the sample coordinate system.

**Supplementary methods**

***Calculation of slip plane normal and slip directions***

Slip plane normal ($\underline{n}_{(hkl)}$) and slip direction ($\underline{v}_{\left( hkl \right)i}$) vectors for the rest of slip systems not detailed in part of ‘Calculation of Schmid factors’ are as follows:

$$\underline{n}_{(010)}=\hat{R}_{Z}\left( 90^{\circ} \right)\cdot\underline{n}_{\left( 100 \right)} with \underline{v}_{\left( 010 \right)i}=\hat{R}_{Z}\left( 90^{\circ} \right)\cdot\underline{v}_{\left( 100 \right)i} i=A,B$$

$$\underline{n}_{(001)}=\hat{R}_{Y}\left( -90^{\circ} \right)\cdot\underline{n}_{\left( 100 \right)} with \underline{v}_{\left( 001 \right)i}=\hat{R}_{Y}\left( -90^{\circ} \right)\cdot\underline{v}_{\left( 100 \right)i} i=A,B$$

$$\underline{n}_{(\bar{1}10)}=\hat{R}_{Z}\left( -90^{\circ} \right)\cdot\underline{n}_{\left( 110 \right)} with \underline{v}_{\left( \bar{1}10 \right)A}=\hat{R}_{Z}\left( -90^{\circ} \right)\cdot\underline{v}_{\left( 110 \right)A}$$

$$\underline{n}_{(10\bar{1})}=\hat{R}_{X}\left( 90^{\circ} \right)\cdot\underline{n}_{\left( 110 \right)} with \underline{v}_{\left( 10\bar{1} \right)A}=\hat{R}_{X}\left( 90^{\circ} \right)\cdot\underline{v}_{\left( 110 \right)A}$$

$$\underline{n}_{(101)}=\hat{R}_{X}\left( 90^{\circ} \right)\cdot\underline{n}_{\left( \bar{1}10 \right)} with \underline{v}_{\left( 101 \right)A}=\hat{R}_{X}\left( 90^{\circ} \right)\cdot\underline{v}_{\left( \bar{1}10 \right)A}$$

$$\underline{n}_{(011)}=\hat{R}_{Z}\left( 90^{\circ} \right)\cdot\underline{n}_{\left( 101 \right)} with \underline{v}_{\left( 011 \right)A}=\hat{R}_{Z}\left( 90^{\circ} \right)\cdot\underline{v}_{\left( 101 \right)A}$$

$$\underline{n}_{(01\bar{1})}=\hat{R}_{Z}\left( 90^{\circ} \right)\cdot\underline{n}_{\left( 10\bar{1} \right)} with \underline{v}_{\left( 01\bar{1} \right)A}=\hat{R}_{Z}\left( 90^{\circ} \right)\cdot\underline{v}_{\left( 10\bar{1} \right)A}$$

$$\underline{n}_{(\bar{1}11)}=\hat{R}_{Z}\left( 90^{\circ} \right)\cdot\underline{n}_{\left( 111 \right)} with \underline{v}_{\left( \bar{1}11 \right)i}=\hat{R}_{Z}\left( 90^{\circ} \right)\cdot\underline{v}_{\left( 111 \right)i} i=A,B,C$$

$$\underline{n}_{(\bar{1}\bar{1}1)}=\hat{R}_{Z}\left( 90^{\circ} \right)\cdot\underline{n}_{\left( \bar{1}11 \right)} with \underline{v}_{\left( \bar{1}\bar{1}1 \right)i}=\hat{R}_{Z}\left( 90^{\circ} \right)\cdot\underline{v}_{\left( \bar{1}11 \right)i} i=A,B,C$$

$$\underline{n}_{(1\bar{1}1)}=\hat{R}_{Z}\left( 90^{\circ} \right)\cdot\underline{n}_{\left( \bar{1}\bar{1}1 \right)} with \underline{v}_{\left( 1\bar{1}1 \right)i}=\hat{R}_{Z}\left( 90^{\circ} \right)\cdot\underline{v}_{\left( \bar{1}\bar{1}1 \right)i} i=A,B,C$$

Where $\hat{R}_{Z}$ and $\hat{R}_{X}$ are identical with that was defined by Eq. (21) and the form of $\hat{R}_{Y}$ rotation is as follows:

$$\hat{R}_{Y}=\left( \begin{matrix} \cos\varphi_{2} & 0 & \sin\varphi_{2} \\ 0 & 1 & 0 \\ -\sin\varphi_{2} & 0 & \cos\varphi_{2} \end{matrix} \right)$$
